# Decreased breast cancer-specific mortality risk in patients with a history of thyroid cancer

**DOI:** 10.1371/journal.pone.0221093

**Published:** 2019-10-23

**Authors:** Weiwei Cheng, Xiaopei Shen, Mingzhao Xing

**Affiliations:** Laboratory for Cellular and Molecular Thyroid Research, Division of Endocrinology, Diabetes & Metabolism, Department of Medicine, Johns Hopkins University School of Medicine, Baltimore, Maryland, United States of America; Kaiser Permanente Washington Health Research Institute, UNITED STATES

## Abstract

Previous studies have documented an intrinsic association between breast cancer (**BC**) and thyroid cancer (**TC**), but the clinical relevance of this relationship is not well defined. In the present study, we specifically investigated the impact of a history of TC on clinical outcomes of BC. We performed a population-based comparative analysis of tumor behaviors and BC-specific mortalities in 427,893 female patients with BC in the USA Surveillance, Epidemiology and End Results 9 database (1973–2013). In this cohort of subjects, 2,569 patients also had a history of differentiated TC (**BC/TC**), including BC diagnosed before TC (**BC-1st**) and BC diagnosed after TC (**TC-1st**), with the median follow-up time of 81 (IQR, 33–160) months. We found that, compared with matched BC-only patients, less aggressive BC tumor behaviors occurred in BC/TC patients, as exemplified by a distant metastasis rate of 7.0% in the former versus 3.3% in the latter (*P*<0.001). In BC/TC, BC-1st, and TC-1st patients versus their matched BC-only patients, BC-specific mortalities were 11.3% versus 21.0%, 9.9% versus 26.4%, and 12.4% versus 16.9%. These corresponded to hazard ratios (HR) (95% CI) of 0.47 (0.42–0.53), 0.31 (0.26–0.37), and 0.72 (0.61–0.84), respectively (all *P*<0.001), being lowest in BC-1st patients <50 years old [HR = 0.22 (0.16–0.31)], which remained significant after adjustment for clinicopathological and socioeconomic factors. Estrogen/progesterone receptor expression in BC tumors was significantly higher in patients with BC/TC than matched BC-only patients, providing evidence that BC in the former was biologically unique. Thus, a history of TC, particularly in younger BC-1st patients, may identify BC as a unique disease entity characterized by a decreased disease-specific mortality risk. The results have potentially important clinical and biological implications for BC in this special patient population and encourage further studies to confirm.

## Introduction

Breast cancer (BC) is the most common malignancy and second leading cause of cancer-related death in women in the United States of America [1–3]. Differentiated thyroid cancer (TC), of which > 85–90% is papillary thyroid cancer (PTC) with the rest being follicular thyroid cancer (FTC), is another common female-dominant malignancy with a female: male ratio of 3–4:1 [2–4]. Numerous studies have widely documented an intrinsic association between the two cancers in a subpopulation of female patients [5–9]. It is thus clinically common to see BC patients with a history of TC. The number of such patients is rising given today’s rising survival patients with BC or TC and the high incidence of either cancer [2–3]. BC-specific mortality remains a major clinical concern today even though it has significantly declined in recent years thanks to early detection and improved treatments [10–12]. Differentiated TC, in contrast, has a generally excellent prognosis with very low mortality [2–4].

Although the intrinsic association of BC with TC has been well known, its clinical relevance and significance are unclear. We hypothesize that BC in this setting may represent a special disease entity with unique clinical outcomes. Establishment of such a special BC entity by demonstrating its unique clinical outcomes would be clinically interesting and important. To this end, in the present study we investigated the clinical outcomes of BC in patients with a history of TC. We focused particularly on the effect of a history of TC on BC-specific patient mortality/survival using the Surveillance, Epidemiology, and End Results 9 (SEER-9) database and unveiled a significant protective effect of the former on the latter.

## Materials and methods

### Data source and cohort selection

With permission, data files and SEER*Stat software (SEER*Stat version 8.3.2) were downloaded from the SEER website. Data were abstracted from the SEER 9 registry database (November, 2015 submission) (https://seer.cancer.gov/data-software/documentation/seerstat/nov2015/), which covered approximately 10% of the US population, had the longest follow-up period (1973–2013). We included only women from SEER histology codes 8010, 8050, 8140, 8141, 8201, 8211, 8401,8480, 8500, 8501, 8503, 8504, 8507, 8510, 8520, 8521, 8522, 8523, 8524, 8530, 8540, 8541, and 8543 for BC and SEER histology codes 8050, 8260, 8290, 8330, 8331, 8332, 8335, 8340, 8341, 8342, 8343, 8344 for differentiated TC, including PTC and FTC. A total of 427,893 patients with BC were identified, among whom 2,569 patients also had a history of differentiated TC (with 87.8% being PTC) (designated as **BC/TC**), with an overall median age of 61 (IQR, 50–72) years and median follow-up time of 81 (IQR, 33–160) months for BC. The **BC/TC** patients consisted of 1,200 BC subjects with subsequent diagnosis of TC (**BC-1st**) and 1,369 TC subjects with subsequent diagnosis of BC (**TC-1st**). Information on some variables was not available for all the years and we only analyzed patients that had the information of interest available.

BC patients without a history of TC (**BC-only**) were randomly selected to correspondingly match BC/TC, BC-1st or TC-1st patients for age at the diagnosis of BC as well as BC incidence densities from the same year. This helped minimize the potential effect of the changing treatment strategy for BC over the years. Specifically, for each consecutive year of follow-up of BC, we randomly selected BC-only patients diagnosed with BC in the same year in which BC in patients with a history of TC was diagnosed at the same age. Sixteen, 12, and 5 cases of BC/TC, BC-1st, and TC-1st patients, respectively, could not be matched and thus removed. The availability of a large number of BC-only patients allowed us to select the BC-only patients 20, 23, and 27 times the case-matched BC/TC, BC-1st, and TC-1st patients, respectively. This random case-matched selection of BC-only patients was repeated three times and overlap of the BC-only patients between any two selections was only around 22–25% (Fig A in S1 File). Fig B in S1 File illustrates a representative perfect match between the accumulated incidence densities of BC in each matched pair. Fig C in S1 File illustrates the chart flow and results of the random case-matched selection, including the number of BC/TC, BC-1st, and TC-1st patients and their corresponding case-matched BC-only patients. All the analyses were performed on data after the matching. Analysis results of data from one of three such selections are presented in the main text of the manuscript. The virtually identical results from the other two selections are presented in Supplemental Data (Figs H–K and Tables C–F in S1 File). We also performed an analysis focused just on white patients, the largest race component of the cohort. As the SEER data is publicly available and does not contain patient identification information, this study did not need institutional review board’s approval; the study was performed following normal professional and ethics standards.

### Survival and follow-up

We used the cause of death and site recode variable in SEER 9 to extract information on the vital status of patients. The SEER*Stat estimated survival time by subtracting the date of the diagnosis of BC from the date of patient death or, in case of no death, the date of last contact. The date of the last contact for all the living patients in this study was within 12 months from 2013 in the MP-SIR and Case Listing session, which provided the most comprehensive clinicopathological information.

### Socioeconomic status (SES) of patients

Socioeconomic information, including zip code, marital status, medical insurance, employment status, education level (at least bachelor degree), and median family income was extracted. The information on marital status, zip code and medical insurance was at individual level for each patient; the information on education, employment and income was aggregated at the census block group level or county level. There were six categories of the insurance status in the SEER database: 1, uninsured; 2, any medicaid; 3, insured; 4, insured, no specifics; 5, insurance status unknown; 6, blank(s). We classified 1 as NO; 2,3,4 as YES; and 5, 6 as information unavailable. Regarding education and income on the multivariate model, the percentages of patients of having at least a bachelor degree and median family income were used, respectively. We used them as continuous variables in the adjustment of the cox model. As people in a census block group or county are usually more homogeneous for these SES factors, aggregate-level SES was used as a surrogate to approximate individual-level SES [13]. SES information was available for patients from 1990, 2000, 2007–2011, 2008–2012 and 2009–2013. As the SES is considered to be relatively stable in 5 years for people, it is reasonable to assume that these statuses were the same in the five-year periods surrounding decennial census years, including 1988–1992 and 1998–2002. Thus, the patients diagnosed in these periods used the SES information of 1990 and 2000 [14]. For patients diagnosed between 2007–2008 or 2009–2013, the SES information of 2007–2011 or 2009–2013 was used. For patients diagnosed in other years, the SES information was not available. For individual patients, only the SES information of the diagnosis year for BC was used.

### Statistical analysis

Categorical data were summarized as frequencies and percentages. Continuous data were not normally distributed in this study and were thus summarized as medians and interquartile ranges (IQR). The χ2 test was used to analyze categorical variables. Wilcoxon-Mann-Whitney test was used to analyze continuous variables. Life-table method was used to determine cumulative mortality (**CM**). Log-rank test was used to construct Kaplan-Meier survival curves. Cox proportional hazard regression analysis was used to adjust covariates and examine hazard ratios (**HR**) of the effect of a history of TC on BC-specific mortality. All *P* values were 2-tailed and a *P<0*.*05* was considered significant. Statistical analyses were performed using SPSS (version 19.0 for windows; SPSS, Armonk, NY) and GraphPad Prism (version 5 for windows; GraphPad Software, San Diego, CA).

## Results

### Relationship between a history of thyroid cancer and tumor behaviors of breast cancer

As summarized in Table 1, there was a general association between a history of TC and less aggressive tumor behaviors and stages of BC. For example, in comparison with matched BC-only patients, BC/TC patients had smaller BC tumor size (*P<0*.*001*), less common lymph node (**LN**) metastasis, distant metastasis, and disease stages III/IV, and lower BC-specific mortality (all *P<0*.*001*). The rate of distant metastasis, an aggressive behavior of BC most commonly associated with patient mortality [10], was 3.3% versus 7.0% in BC/TC versus BC-only patients (*P<0*.*001*). BC-specific mortality was 11.3% versus 21.0% in BC/TC versus matched BC-only patients (*P<0*.*001*). Positivity of expression of estrogen receptor (**ER**) and progesterone receptor (**PR**) was more common in BC/TC than matched BC-only patients (*P = 0*.*001* for ER and *P = 0*.*004* for PR). The difference between BC-1st and matched BC-only was even more dramatic, as exemplified by the distant metastasis rate and BC-specific mortality of 3.1% and 9.9% versus 7.0% and 26.4% in the former versus the latter, respectively (all *P<0*.*001*). This pattern was also seen with the ER/PR expression positivity. A significant, albeit less, difference in BC-specific mortality was also seen between TC-1st and matched BC-only patients. Several SES factors, such as medical insurance, employment, education level of at least bachelor degree, and median family income were not different between BC patients with TC and BC-only patients.

**Table 1:** Comparison of clinicopathological characteristics of breast cancer in various clinical settings.

	Comparison 1			Comparison 2			Comparison 3		
	BC/TC n/N(%)	BC-only n/N(%)	P value	BC-1st n/N(%)	BC-only n/N(%)	P value	TC-1st n/N(%)	BC-only n/N(%)	P value
Number of cases	2553	51060		1188	27324		1364	36828	
BC diagnosis pre-1990	480	9600		321	7383		159	4293	
BC diagnosis after1990	2073	41460		867	19941		1205	32535	
Age at diagnosis(yrs), Median (IQR)	57 (48–67)	57 (48–67)	1.000	54 (46–63)	54 (46–63)	1.000	60 (51–70)	60 (51–70)	1.000
Tumor Size(mm),Median (IQR)	16 (10–25)	17 (11–28)	<0.001	16 (10–25)	18 (11–28)	0.001	15 (10–25)	17 (10–27)	<0.001
Pathology Ductal	2044/2553 (80.1)	40706/51060 (79.7)	0.685	969/1188 (81.6)	21918/27324 (80.2)	0.252	1074/1364 (78.7)	29156/36828 (79.2)	0.711
Lobular	208/2553 (8.1)	3927/51060 (7.7)	0.404	86/1188 (7.2)	1876/27324 (6.9)	0.619	122/1364 (8.9)	3102/36828 (8.4)	0.490
Mixed	212/2553 (8.3)	4072/51060 (8.0)	0.552	83/1188 (7.0)	1906/27324 (7.0)	0.988	129/1364 (9.5)	3195/36828 (8.7)	0.305
Inflammatory	11/2553 (0.4)	328/51060 (0.6)	0.251	6/1188 (0.5)	199/27324 (0.7)	0.373	5/1364 (0.4)	207/36828 (0.6)	0.456
ER/PR status ER-positive	1551/1901 (81.6)	30135/38501 (78.3)	0.001	628/788 (79.7)	13785/18337 (75.2)	0.004	922/1112 (82.9)	24421/30466 (80.2)	0.025
PR-positive	1334/1877 (71.1)	25941/38194 (67.9)	0.004	548/776 (70.6)	11890/18144 (65.5)	<0.001	785/1100 (71.4)	20955/30251 (69.3)	0.144
Both positive	1299/1876 (69.2)	25126/38161 (65.8)	0.002	528/776 (68.0)	11424/18130 (63.0)	0.005	770/1099 (70.1)	20443/30241 (67.6)	0.088
LN metastasis	737/2277 (32.4)	16323/45201 (36.1)	<0.001	333/1009 (33.0)	8751/22802 (38.4)	0.001	404/1267 (31.9)	11767/34207 (34.4)	0.067
Distant Metastasis	82/2516 (3.3)	3491/50134 (7.0)	<0.001	36/1168 (3.1)	1864/26726 (7.0)	<0.001	46/1347 (3.4)	2438/36284 (6.7)	<0.001
AJCC Stage I+II	1762/2059 (85.6)	33648/41535 (81.0)	<0.001	734/862 (85.2)	16089/20259 (79.4)	<0.001	1027/1196 (85.9)	26406/32267 (81.8)	<0.001
III+IV	297/2059 (14.4)	7887/41535 (19.0)		128/862 (14.8)	4170/20259 (20.6)		169/1196 (14.1)	5861/32267 (18.2)	
Radiation Therapy	1211/2498 (48.5)	25008/49691 (50.3)	0.074	555/1167 (47.6)	12688/26648 (47.6)	0.970	656/1330 (49.3)	18681/35823 (52.1)	0.044
BC-specific Mortality	288/2553 (11.3)	10743/51060 (21.0)	<0.001	118/1188 (9.9)	7217/27324 (26.4)	<0.001	169/1364 (12.4)	6218/36828 (16.9)	<0.001
Follow Up Time; Median (IQR)	102 (44–188)	80 (35–156)	<0.001	155 (82–240)	102 (49–188)	<0.001	65 (29–135)	63 (26–130)	0.152
With Insurance	801/809 (99.0)	15987/16182 (98.8)	0.699	225/229 (98.3)	5149/5214 (98.8)	0.719	575/579 (99.3)	15525/15712 (98.8)	0.3683
Married	1629/2455 (66.4)	30217/49129 (61.5)	<0.001	784/1142 (68.7)	16951/26390 (64.2)	0.003	845/1312 (64.4)	21028/35263 (59.6)	<0.001
Unemployed*	115/1513 (7.6)	2335/30244 (7.7)	0.904	40/575 (7.0)	945/13217 (7.1)	0.926	74/937 (7.9)	2048/25286 (8.1)	0.872
Education (at least bachelor degree)*	467/1513 (30.9)	9439/30244 (31.2)	0.800	173/575 (30.1)	3988/13217 (30.2)	0.971	294/937 (31.4)	8043/25286 (31.8)	0.808
Median family income (US dollar); median (IQR)	65540 (51440–80740)	65860 (51650–80620)	0.863	60550 (46610–79600)	60550 (48010–75540)	0.740	66610 (53000–82690)	66610 (53000–81810)	0.868

**Abbreviations**: BC, breast cancer; TC, thyroid cancer; BC-only, patients only with breast cancer and without a history of thyroid cancer; BC/TC, breast cancer patients also with a history of thyroid cancer diagnosed any time—either before or after the diagnosis of breast cancer; BC-1st, breast cancer was diagnosed first, followed by subsequent diagnosis of thyroid cancer; TC-1st, thyroid cancer was diagnosed first, followed by subsequent diagnosis of breast cancer; IQR, interquartile range; ER, estrogen receptor; PR, progesterone receptor; LN, lymph node. *The numbers of unemployed cases and cases with an education of least bachelor degree were obtained by calculation based on the unemployment rates and rates of education of at least bachelor degree and the corresponding total cases, respectively, in different counties.

White people accounted for 75.9%-79.5% of patients in different groups. Virtually identical results were obtained when the analysis was focused on the white patients (Table A in S1 File). For example, BC-specific mortality was 11.4% versus 20.7%, 10.6% versus 25.8%, and 12.1% versus 16.5% in BC/TC, BC-1st, and TC-1st versus their corresponding case-matched BC-only patients, respectively (all *P<0*.*001*). Expression positivity of ER and PR in BC was also more common in patients with a history of TC than matched BC-only patients.

### Relationship between a history of thyroid cancer and breast cancer-specific cumulative mortality/survival of patients

The BC-specific CMs of BC/TC versus BC-only patients were 6.2% versus 15.3% at 5 years, 11.3% versus 23.2% at 10 years, 15.1% versus 28.0% at 15 years, and 18.3% versus 31.9% at 20 years, respectively (all *P <0*.*001*); the BC-specific CMs of BC-1st versus matched BC-only patients was 3.1% versus 16.7% at 5 years, 7.1% versus 25.7% at 10 years, 10.1% versus 30.6% at 15 years, and 13.4% versus 44.0% at 20 years, respectively (all *P <0*.*001*); and the BC-specific CMs of TC-1st versus matched BC-only patients was 9.3% versus 14.2% at 5 years, 16.1% versus 21.5% at 10 years, 21.6% versus 26.4% at 15 years, and 24.7% versus 30.0% at 20 years, respectively (all *P <0*.*001*). This consistent pattern of lower BC-specific CM in patients with a history of TC, particularly prominently in BC-1st patients, is better seen in Fig D in S1 File. Similar patterns were seen with the cumulative overall mortality except for an insignificant difference in the overall mortality between TC-1st and matched BC-only patients (Fig D in S1 File). Virtually identical CM results were obtained when only white patients were analyzed (Fig E in S1 File). As summarized in Table 2, BC-specific deaths per 1000 person-years were 10.7 (95% CI, 9.5–12.0) versus 23.8 (95% CI, 23.3–24.2) in BC/TC versus BC-only patients, with a HR of 0.47 (95% CI, 0.42–0.53; *P<0*.*001*), which remained significant at 0.55 (95% CI, 0.43–0.70; *P<0*.*001*) after multivariate adjustment for race, tumor size, pathological type, ER/PR status, LN metastasis, radiation therapy, marital status, employment status, education level of at least bachelor degree, and median family income. Deaths per 1000 person-years were 7.1 (95% CI, 5.8–8.5) versus 26.4 (95% CI, 25.8–27.0) in BC-1st versus BC-only patients, with a HR of 0.31 (95% CI, 0.26–0.37; *P<0*.*001*), which remained significant at 0.30 (95% CI, 0.19–0.48; *P<0*.*001*) after the above multivariate adjustment. Deaths per 1000 person-years were 16.6 (95% CI, 14.2–19.3) versus 23.2 (95% CI, 22.7–23.8) in TC-1st versus BC-only patients, with a HR of 0.72 (95% CI, 0.61–0.84; *P<0*.*001*), which became insignificant after multivariate adjustments.

**Table 2:** Effects of a history of thyroid cancer on breast cancer-specific mortality—Deaths per 1000 person-years and hazard ratios.

		BC-specific Mortality, n/N (%)	Deaths per 1000 Person-Years (95% CI)	Unadjusted		Adjustment^a^		Adjustment^b^	
				HR (95% CI)	P value	HR (95% CI)	P value	HR (95% CI)	P value
Comparison 1	BC-only	10743/51060 (21.0)	23.8 (23.3–24.2)	1.000		1.000		1.000	
	BC/TC	288/2553 (11.3)	10.7 (9.5–12.0)	0.47 (0.42–0.53)	<0.001	0.67 (0.56–0.80)	<0.001	0.55 (0.43–0.70)	<0.001
Comparison 2	BC-only	7217/27324 (26.4)	26.4 (25.8–27.0)	1.000		1.000		1.000	
	BC-1st	118/1188 (9.9)	7.1 (5.8–8.5)	0.31 (0.26–0.37)	<0.001	0.46 (0.35–0.62)	<0.001	0.30 (0.19–0.48)	<0.001
Comparison 3	BC-only	6218/36828 (16.9)	23.2 (22.7–23.8)	1.000		1.000		1.000	
	TC-1st	169/1364 (12.4)	16.6 (14.2–19.3)	0.72 (0.61–0.84)	<0.001	0.88 (0.71–1.10)	0.264	0.78 (0.59–1.03)	0.084

Adjustment^a^ was made for race, tumor size, pathological type, ER/PR status, LN metastasis, radiation therapy, marital status and zip code.

Adjustment^b^ was made for race, tumor size, pathological type, ER/PR status, LN metastasis, radiation therapy, married status, employment status, education (at lease bachelor degree), and median family income.

Abbreviations: BC, breast cancer; TC, thyroid cancer; BC-only, patients only with breast cancer and without a history of thyroid cancer; BC/TC, breast cancer patients also with a history of thyroid cancer diagnosed any time—either before or after the diagnosis of breast cancer; BC-1st, breast cancer was diagnosed first, followed by subsequent diagnosis of thyroid cancer; TC-1st, thyroid cancer was diagnosed first, followed by subsequent diagnosis of breast cancer; CI, confidence interval; ER, estrogen receptor; PR, progesterone receptor; LN, lymph node.

**Fig 1:**
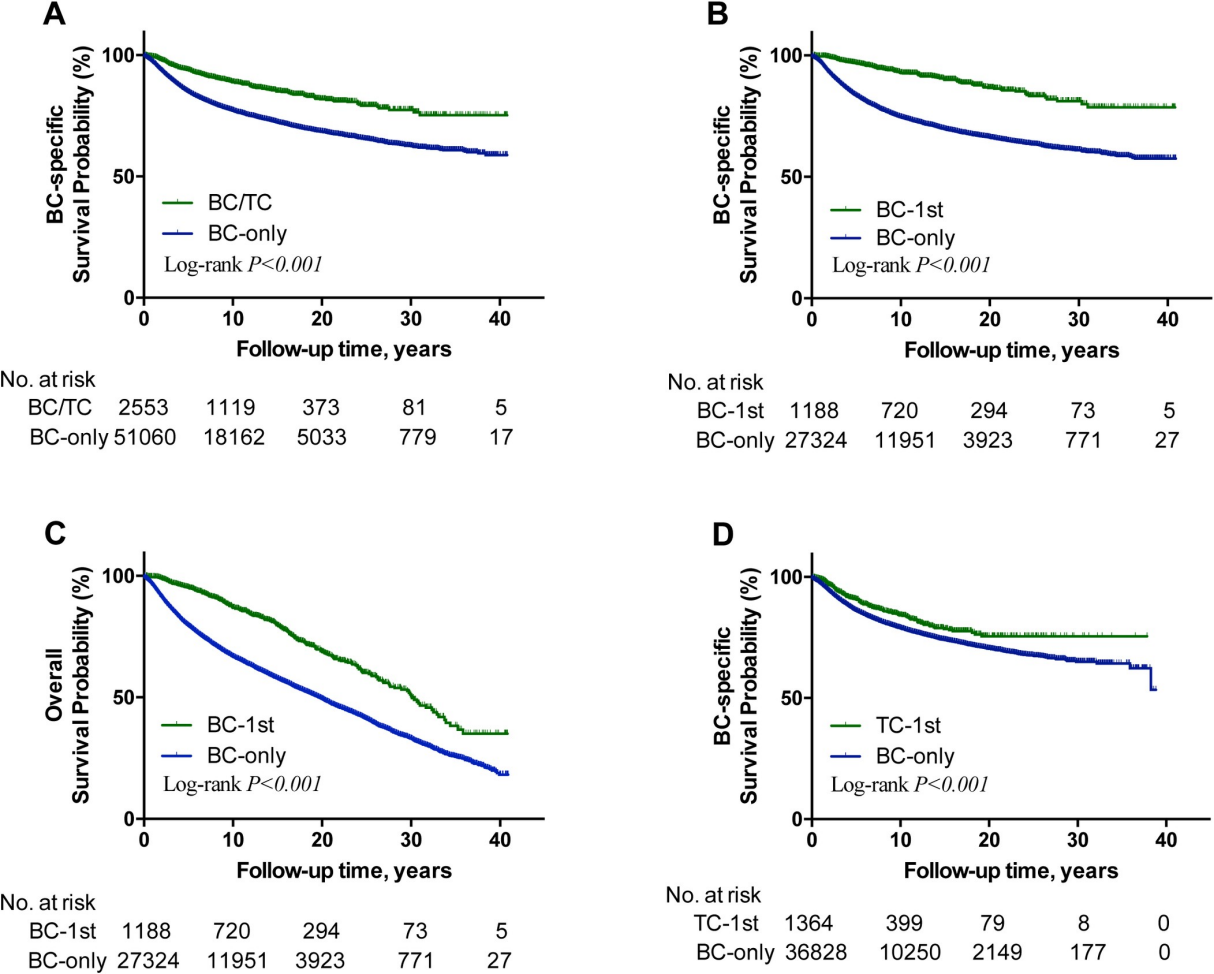
Kaplan-Meier analysis of the effect of a history of TC on BC-specific and overall survivals of patients in various settings. **A**, comparison of BC-specific survival curves between BC/TC and matched BC-only patients; **B**, comparison of BC-specific survival curves between BC-1st and matched BC-only patients; **C**, comparison of overall survival curves between BC-1st and matched BC-only patients. **D**, comparison of BC-specific survival curves between TC-1st and matched BC-only patients. **BC**, breast cancer; **TC**, thyroid cancer; **BC-only**, patients only with a diagnosis of breast cancer and without a history of thyroid cancer; **BC/TC**, breast cancer patients also with a history of thyroid cancer diagnosed any time—either before or after the diagnosis of breast cancer; **BC-1st**, breast cancer was diagnosed first, followed by the diagnosis of thyroid cancer; **TC-1st**, thyroid cancer was diagnosed first, followed by the diagnosis of breast cancer.

Kaplan-Meier analyses revealed a much slower decline of BC-specific and overall survival curves in BC/TC (Fig 1A and Fig F in S1 File, respectively) and BC-1st (Fig 1B and 1C, respectively) patients compared with their matched BC-only patients (all *P<0*.*001*). A slower decline was also seen in BC-specific survival curve (Fig 1D), but not the overall survival curve (Fig F in S1 File), in TC-1st patients compared with the matched BC-only patients. Virtually identical results were obtained in the analyses of only white patients (Fig G and Table B in S1 File).

The above results were from patients in random case-matched selection one. Virtually identical results were achieved on the analyses of patients in random case-matched selection two (Figs H-I and Tables C-D in S1 File) and selection three (Figs J-K and Tables E-F in S1 File).

### Effect of a history of thyroid cancer on breast cancer-specific survival in younger patients

**Fig 2:**
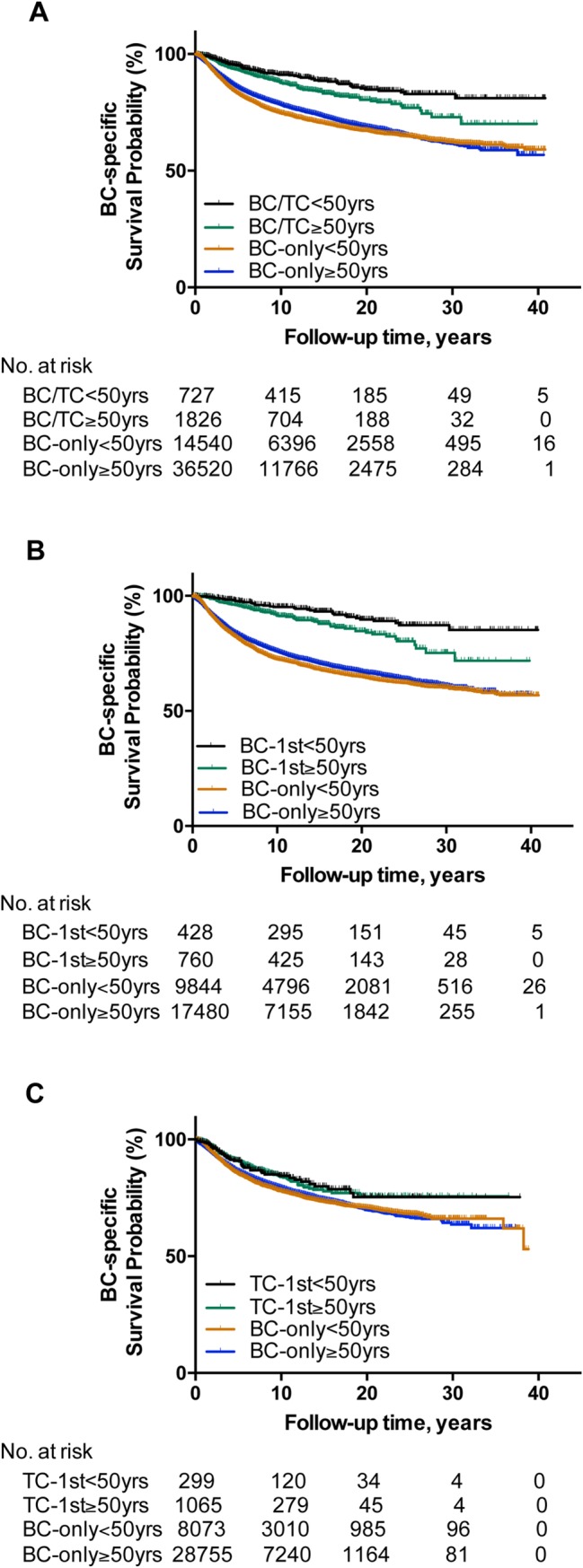
Kaplan-Meier analyses of the differential protective effects of a history of TC on BC-specific survival between young and old patients. **A**, comparisons of the effects of a history of TC on BC-specific survival curves in BC/TC and matched BC-only patients between the group aged < 50 years and the group aged ≥ 50 years. **B**, comparisons of the effects of a history of TC on BC-specific survival curves in BC-1st and matched BC-only patients between the group aged < 50 years and the group aged ≥ 50 years. **C**, comparisons of the effects of a history of TC on BC-specific survival curves in TC-1st and matched BC-only patients between the group aged < 50 years and the group aged ≥ 50 years. **BC**, breast cancer; **TC**, thyroid cancer; **BC-only**, patients only with a diagnosis of breast cancer and without a history of thyroid cancer; **BC/TC**, breast cancer patients also with a history of thyroid cancer diagnosed any time—either before or after the diagnosis of breast cancer; **BC-1st**, breast cancer was diagnosed first, followed by diagnosis of thyroid cancer; **TC-1st**, thyroid cancer was diagnosed first, followed by diagnosis of breast cancer.

We observed a generally stronger protective effect of a history of TC on BC-specific survival in younger patients when dividing the patients into age groups of <50 years and ≥50 years (Table 3). Specifically, HRs (95% CI) for the effect of a history of TC on BC-specific mortality in BC/TC, BC-1st, TC-1st were 0.37 (0.29–0.46), 0.22 (0.16–0.31), and 0.72 (0.53–0.97) in patients aged <50 years and 0.53 (0.47–0.61), 0.37 (0.30–0.46), and 0.72 (0.60–0.86) in patients aged ≥50 years, respectively. These became 0.44 (0.27–0.72), 0.21 (0.09–0.51), and 0.87 (0.49–1.55) in patients aged <50 years and 0.58 (0.44–0.77), 0.35 (0.21–0.59), and 0.73 (0.52–1.01) in patients aged ≥50 years, respectively, after multivariate adjustment. Among all these settings, the protective effect of a history of TC in BC-1st patients aged <50 years was most robust. These patterns of the protective effects of a history of TC were also reflected in the BC-specific survival curves, showing a separation between the younger and older patients with BC/TC (Fig 2A) or BC-1st (Fig 2B), but not TC-1st (Fig 2C) patients. The survival curve in the BC-1st patients aged <50 years had the slowest decline (Fig 2B). In all these settings, the decline in the BC-specific survival curve was slower in patients with a history of TC than the matched BC-only patients.

**Table 3:** Differential protective effects of a history of thyroid cancer on breast cancer-specific mortality in patients at age<50years and age≥50years at the diagnosis of breast cancer.

		BC-specific Mortality	Deaths per 1000 Person-Years (95% CI)	Unadjusted		Adjustment^a^		Adjustment^b^	
				HR (95% CI)	P Value	HR (95% CI)	P Value	HR (95% CI)	P Value
Comparison 1 Age<50yrs	BC-only	3730/14540 (25.7)	23.5 (22.7–24.2)	1.000		1.000		1.000	
	BC/TC	79/727 (10.9)	7.9 (6.3–9.9)	0.37 (0.29–0.46)	<0.001	0.50 (0.35–0.71)	<0.001	0.44 (0.27–0.72)	<0.001
Comparison 1 Age> = 50yrs	BC-only	7013/36520 (19.2)	24.0 (23.4–24.5)	1.000		1.000		1.000	
	BC/TC	209/1826 (11.4)	12.3 (10.7–14.1)	0.53 (0.47–0.61)	<0.001	0.75(0.61–0.92)	0.006	0.58 (0.44–0.77)	<0.001
Comparison 2 Age<50yrs	BC-only	2912/9844 (29.6)	24.5 (23.6–25.4)	1.000		1.000		1.000	
	BC-1st	34/428 (7.9)	4.8 (3.3–6.7)	0.22 (0.16–0.31)	<0.001	0.33 (0.19–0.57)	<0.001	0.21 (0.09–0.51)	<0.001
Comparison 2 Age> = 50yrs	BC-only	4305/17480 (24.6)	25.2 (24.4–26.0)	1.000		1.000		1.000	
	BC-1st	84/760 (11.1)	8.7 (6.9–10.7)	0.37 (0.30–0.46)	<0.001	0.49 (0.34–0.70)	<0.001	0.35 (0.21–0.59)	<0.001
Comparison 3 Age<50yrs	BC-only	1639/8073 (20.3)	21.7 (20.6–22.7)	1.000		1.000		1.000	
	TC-1st	45/299 (15.1)	15.5 (11.3–20.7)	0.72 (0.53–0.97)	0.028	1 (0.67–1.49)	1	0.87 (0.49–1.55)	0.638
Comparison 3 Age> = 50yrs	BC-only	4579/28755 (15.9)	23.8 (23.2–24.5)	1.000		1.000		1.000	
	TC-1st	124/1065 (11.6)	17.0 (14.1–20.3)	0.72 (0.60–0.86)	<0.001	0.90 (0.70–1.16)	0.427	0.73(0.52–1.01)	0.056

Adjustment^a^ was made for race, tumor size, pathology type, ER/PR status, LN metastasis, distant metastasis, radiation therapy, marital status and zip code.

Adjustment^b^ was made for race, tumor size, pathology type, ER/PR status, LN metastasis, distant metastasis, radiation therapy, marital status, employment status, education level (at lease bachelor degree), and median family income.

Abbreviations: BC, breast cancer; TC, thyroid cancer; BC-only, patients only with breast cancer without a history of thyroid cancer; BC/TC, breast cancer patients also with a history of thyroid cancer diagnosed any time—either before or after the diagnosis of breast cancer; BC-1st, breast cancer was diagnosed first, followed by subsequent diagnosis of thyroid cancer; TC-1st, thyroid cancer was diagnosed first, followed by subsequent diagnosis of breast cancer; CI, confidence interval; ER, estrogen receptor; PR, progesterone receptor; LN, lymph node.

PTC is the most common TC and accounted for the vast majority of the cases of TC in this study. A PTC patient-focused analysis revealed virtually identical protective effects of a history of PTC on BC-specific mortalities (Figs L-N and Tables G-I in S1 File). There was too small a number of FTC patients to conduct a FTC-focused analysis.

## Discussion

Although previous studies have widely observed and established an intrinsic association between BC and TC [5–9], the clinical relevance and significance of this relationship, particularly in terms of impact on clinical outcomes of BC, have not been well investigated. With our hypothesis that BC in patients with a history of TC may represent a unique disease entity, we for the first time investigated the effect of a history of TC on BC-specific patient mortality/survival. We chose to do so given the well-known extremely low TC-specific mortality but much higher BC-specific mortality.

We observed a strong protective effect of a history of TC on BC-specific patient survival. This protective effect was particularly robust in BC-1st patients, with a BC-specific mortality HR of 0.31 (95% CI 0.26–0.37), which was even more dramatic at 0.22 (95% CI 0.16–0.31) in patients <50 years old. These HRs remained highly significant even after multivariate adjustment for clinicopathological and SES factors. To be consistent with this protective effect of a history of TC on BC-specific patient survival were the generally low-grade initial BC tumor behaviors in patients with a history of TC, as exemplified by a significantly lower distant metastasis rate compared with BC-only patients (Table 1).

Several important aspects of this study deserve a special discussion. 1) The SEER data were collected over several decades. As such, patient age and treatment variations over the years may affect the clinical outcomes of BC. This was effectively eliminated or minimized by our case-matched selection strategy to match the patient age at the diagnosis of BC and, importantly, match also incidence densities of BC in each consecutive individual year for matched BC-only patients and BC patients with a history of TC. To be consistent with this conclusion is that the results from three such random case- and incidence density-matched selections were virtually identical. 2) Diagnosis of TC may promote clinical surveillance, leading to early detection and treatment of BC with better clinical outcomes, which could cause an apparent “protective effect” of TC. This, however, could potentially occur only in TC-1st patients, but not in BC-1st patients. In fact, the protective effect of TC on BC-specific survival was more robust in BC-1st patients than TC-1st patients. 3) It is possible that in BC-1st patients it takes time to diagnose subsequent TC and, consequently, patients “living longer to wait for the occurrence of TC” are selected. This was very unlikely the case in this study, however; the median latency period between the diagnosis of BC and subsequent diagnosis of TC was 5.2 (IQR, 1.3–11.2) years, while the median follow-up time of BC-only patients was 6.8 (IQR, 2.8–13.4) years, with the former actually being shorter than the latter. Thus, this type of immortal time bias may not explain the protective effect seen in the BC-first patients. Nevertheless, some effect of immortal time bias cannot be completely ruled out. 4) It is interesting that the protective effect of TC on patient survival was relatively modest in TC-1st patients compared with BC-1st patients. One explanation is the much shorter follow-up time of BC in the former (Table 1) because their BC was diagnosed in late years following the diagnosis of TC first as shown in Fig B in S1 File. Since BC-specific death takes time to occur, the short follow-up time of BC may not be sufficient to allow for the protective effect of a history of TC to express. It is also possible that young patient age synergizes the protective effect of a history of TC since this effect was most robust in younger patients (Table 3 and Fig 2). This speculation is reasonable given the fact that old patient age is generally a high risk for poor prognosis of human cancers. This is consistent with the fact that TC-1st patients were much older than BC-1st patients at the diagnosis of BC (median age of TC-1st patients versus BC-1st patients was 60 years versus 54 years, *P<0*.*001*). It is possible that in the younger BC-1st patients, who are mostly in the premenopausal status, estrogen and progesterone are naturally at higher levels than in older TC-1st patients, who are mostly in the postmenopausal status at the diagnosis of BC, thus making the expression of ER/PR functionally more meaningful and hormonally modulatory adjuvant treatment of BC more effective in the former. Another interesting possibility is that as a biologically unique and intrinsically benign-prognostic BC entity identified by a history of TC (see below for further discussion), it is possible that BC in this setting may naturally tend to develop earlier than TC in the patient. 5) Patients with BC often have increased screening/surveillance imaging studies which tend to reveal thyroid nodules and lead to increased diagnosis of TC, raising the question of whether this is why BC in patients with a history of TC has a better prognosis. This is not the case as BC in such a setting would likely have a poor prognosis because data have shown that it is those patients with relatively high-grade BC who more often use screening/surveillance imaging studies and would therefore potentially have increased diagnosis of TC [15]. As such, BC in these patients with an associated history of TC would in fact be expected to have a poorer prognosis. Nevertheless, our findings require further studies to confirm given the potential influence of complex confounding factors.

It is possible that BC in patients with a history of TC represents a unique disease entity that is determined by an intrinsic biological background. It is likely a unique genetic background, as exemplified by the concurrence of BC and TC in certain tumor syndromes, such as the *PTEN* gene defect-associated Cowden Syndrome, in which both PTC and FTC can occur albeit with the latter being more common than the former [16,17]. This hypothesis is consistent with the finding of increased concurrence of BC and TC in first-degree relatives of probands with either cancer [18]. Importantly, the biological uniqueness of BC in patients with a history of TC is directly supported by the finding of more common ER/PR expression positivity in this entity of BC than BC in patients without a history of TC (Table J in S1 File). In fact, a higher ER/PR positivity rate was seen particularly in BC-1st patients <50 years old, consistent with the best protective effect of a history of TC observed in this setting of patients. Two recent studies reported similarly higher expression positivity of ER/PR in BC in patients with a history of TC [5,8]. The results on ER/PR positivity in the SEER database are not quantitatively stratified; cases of BC with low level of detectable expression of ER/PR may be treated as positive for ER/PR. It is thus possible, albeit requiring to be proven, that not only the rate of ER/PR positivity, as currently defined in the SEER database, is higher but the expression level may also be quantitatively higher in BC in patients with a history of TC than patients with BC-only. In other words, the actual difference in ER/PR expression in BC between patients with a history of TC and patients without TC may be even more pronounced than the apparent difference reported in the present study. Regardless, the ER/PR results provide biological evidence that BC in patients with a history of TC represents a biologically unique disease entity. They also provide a direct biological explanation for the low mortality of this unique BC entity since positive ER/PR is well known to be associated with a better prognosis of BC [19,20].

In summary, this large comprehensive study suggests a strong protective effect of a history of TC on BC-specific patient survival, particularly in younger BC-1st patients, who have a significantly lower mortality than BC-only patients. BC in these patients may represent a special disease entity that is clinically and biologically unique; it is characterized by decreased risk of disease-specific mortality and higher ER/PR expression. The results have potentially important clinical and biological implications for BC in this special patient population and encourage further clinical and biological studies.

## Supporting information

S1 File Cheng et al: Decreased breast cancer-specific mortality risk in patients with a history of thyroid cancer. (DOCX)
