# Correction: Decreased breast cancer-specific mortality risk in patients with a history of thyroid cancer

**DOI:** 10.1371/journal.pone.0225986

**Published:** 2019-11-26

**Authors:** Weiwei Cheng, Xiaopei Shen, Mingzhao Xing

There are errors in the Author Contributions. The correct contributions are:

**Conceptualization:** Mingzhao Xing

**Data curation:** Weiwei Cheng, Xiaopei Shen, Mingzhao Xing

**Formal analysis:** Weiwei Cheng, Xiaopei Shen, Mingzhao Xing

**Funding acquisition:** Mingzhao Xing

**Methodology:** Xiaopei Shen

**Project administration:** Mingzhao Xing

**Supervision:** Mingzhao Xing

**Validation:** Weiwei Cheng, Xiaopei Shen, Mingzhao Xing

**Visualization:** Weiwei Cheng, Xiaopei Shen, Mingzhao Xing

**Writing–original draft:** Weiwei Cheng, Xiaopei Shen, Mingzhao Xing

**Writing–review & editing:** Mingzhao Xing
