# Supplementary material for: Decreased breast cancer-specific mortality risk in patients with a history of thyroid cancer
Source: PLoS One. 2019 Oct 23;14(10):e0221093. doi: 10.1371/journal.pone.0221093 (PMC6808426; doi:10.1371/journal.pone.0221093)
Supplement: S1 File — (DOCX) [file pone.0221093.s001.docx]

**CONTENTS**

Fig A. Venn diagram illustration of overlap among the matched BC-only patients on three repeated case-match selections for BC patients with a history of TC 3

Fig B. Case match of accumulated incidence densities of BC between patients with BC only and patients also with a history of TC.4

Fig C. The flow chart and results of case-matched selection of BC-only patients.5

Fig D. Comparison of the cumulative mortalities of patients between matched BC-only patients and BC patients also with a history of TC.6

Fig E. Comparison of the cumulative mortalities of patients between matched BC-only patients and BC patients also with a history of TC in white people.7

Fig F. Kaplan-Meier analysis of the effect of a history of TC on overall survivals of patients in BC/TC and TC-1st patients.8

Fig G. Kaplan-Meier analysis of the effect of a history of TC on BC-specific and overall survivals of white patients in various settings.9

Fig H. Comparison of the cumulative mortalities of patients between BC patients with a history of TC and matched patients only with BC (data from the second random case selection).10

Fig I. Kaplan-Meier analysis of the effect of a history of TC on BC-specific and overall survivals of patients (data from the second random case selection).11

Fig G. Comparison of the cumulative mortalities of patients between BC patients with a history of TC and matched patients only with BC (data from the third random case selection).12

Fig K. Kaplan-Meier analysis of the effect of a history of TC on BC-specific and overall survivals of patients (data from the third random case selection).13

Fig L. Comparison of the cumulative mortalities between breast cancer patients with a history of papillary thyroid cancer and matched patients only with breast cancer..14

Fig M. Kaplan-Meier analysis of the effects of a history of papillary thyroid cancer on breast cancer-specific and overall survivals of patients in various settings..15

Fig N. Kaplan-Meier analyses of the differential protective effects of a history of papillary thyroid cancer on breast cancer-specific survival between young and old patients16

Table A. Comparison of clinicopathological characteristics of breast cancer in various clinical settings in white people 17

Table B. Effects of a history of thyroid cancer on breast cancer-specific mortality—deaths per 1000 person-years and hazard ratios in white people.19

Table C. Comparison of clinicopathological characteristics of breast cancer in various clinical settings (data from the second case selection).20

Table D. Effects of a history of thyroid cancer on breast cancer-specific mortality—deaths per 1000 person-years and hazard ratios (data from the second case selection).22

Table E. Comparison of clinicopathological characteristics of breast cancer in various clinical settings (data from the third case selection).23

Table F. Effects of a history of thyroid cancer on breast cancer-specific mortality—deaths per 1000 person-years and hazard ratios (data from the third case selection).25

Table G. Comparison of clinicopathological characteristics of breast cancer between patients also with a history of papillary thyroid cancer and patients only with breast cancer.26

Table H. Effects of a history of papillary thyroid cancer on breast cancer-specific mortality.28

Table I. Differential protective effects of a history of papillary thyroid cancer on breast cancer-specific mortality in patients at age<50years and age≥50years at the diagnosis of breast cancer.29

Table J. Comparison of ER/PR expression status in breast cancer between patients also with a history of thyroid cancer and patients only with breast cancer at age <50 years or age ≥50 years30

**Fig A. Venn diagram illustration of overlap among the age- and incidence density-matched BC-only patients on three repeated case-match selections for BC patients with a history of TC.**

**
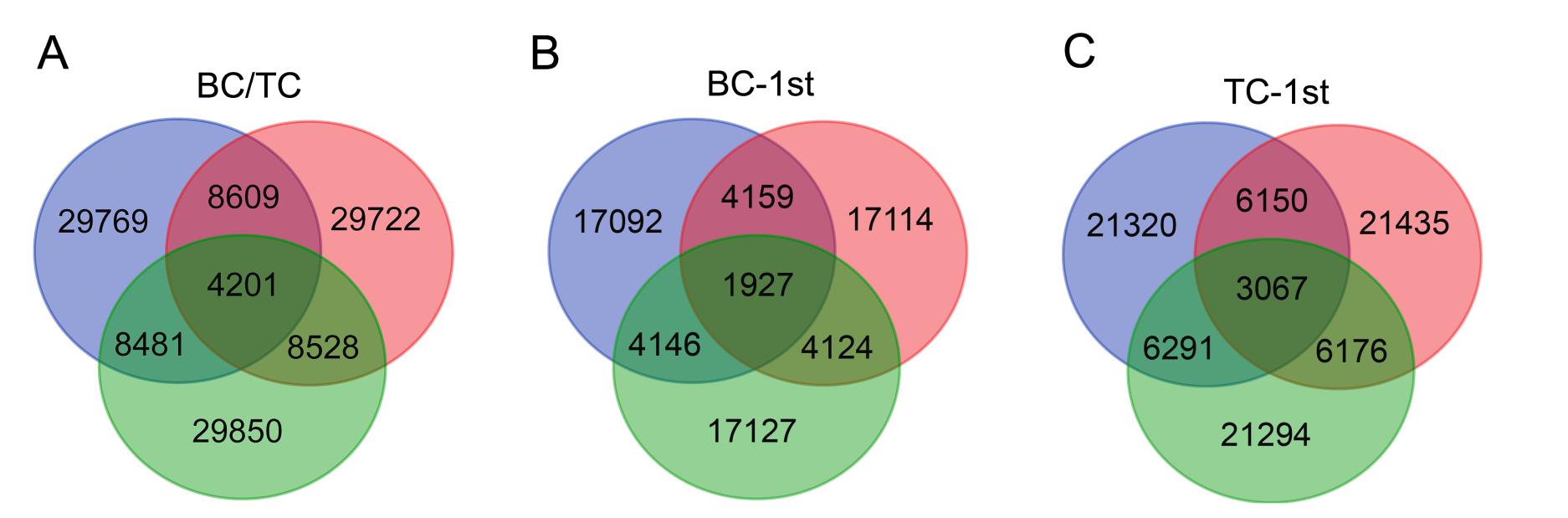
**

**A**, shown is the overlap of BC-only patients among the three times of match for BC/TC patients. The overlap between any two times of match ranged from 24.8% to 25.1%. **B**, shown is the overlap of BC-only patients in the three times of match for BC-1st patients. The overlap between any two times of match ranged from 22.1% to 22.3%. **C**, shown is the overlap of BC-only patients in the three times of match for TC-1st patients. The overlap between any two times of match ranged from 25.0% to 25.4%. **BC**, breast cancer; **TC**, thyroid cancer; **BC-only**, patients only with a diagnosis of breast cancer without a history of thyroid cancer; **BC/TC**, breast cancer patients also with a history of thyroid cancer diagnosed any time—either before or after the diagnosis of breast cancer; **BC-1st**, breast cancer was diagnosed first, followed by diagnosis of thyroid cancer; **TC-1st**, thyroid cancer was diagnosed first, followed by diagnosis of breast cancer.

**Fig B**. **Perfect case match of accumulated incidence densities of BC between patients with BC only and patients also with a history of TC**


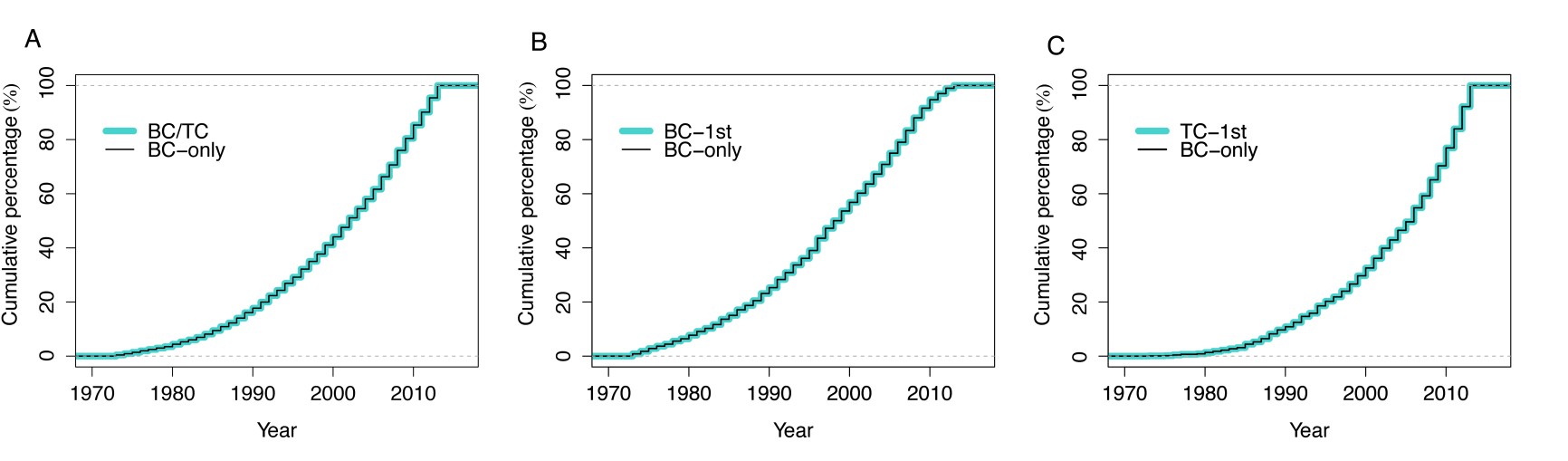


For each consecutive year of follow-up, patients only with BC diagnosed in the same year in which BC in diagnosis age-matched patients with a history of TC was diagnosed were randomly selected. Sufficient BC-only patients available allowed choosing at 20, 23, and 27 times the age-matched BC/TC, BC-1st, and TC-1st patients in each year, respectively. The incidence densities are represented by the accumulated percentage of cases (the Y-axis) in corresponding individual years of the follow-up (the X-axis) of the entire case cohort included in the study; the accumulated incidence densities were 0% at the beginning and 100% at the end of the final follow-up year. **A**, shown is the incidence density match of BC between patients with BC only and patients also with a history of TC diagnosed any time. **B**, shown is the incidence density match of BC between patients with BC only and patients also with a history of TC diagnosed after the diagnosis of BC. **C**, shown is the incidence density match of BC between patients with BC only and patients also with a history of TC diagnosed before the diagnosis of BC. The blue line represents BC patients with a history of TC and the black line represents BC patients without a history of TC. In each panel, the two lines of BC of the paired patients completely overlap, representing a perfect match. This represents one of the three random case-matched selections. **BC**, breast cancer; **TC**, thyroid cancer; **BC-only**, patients only with a diagnosis of breast cancer without a history of thyroid cancer; **BC/TC**, breast cancer patients also with a history of thyroid cancer diagnosed any time—either before or after the diagnosis of breast cancer; **BC-1st**, breast cancer was diagnosed first, followed by diagnosis of thyroid cancer; **TC-1st**, thyroid cancer was diagnosed first, followed by diagnosis of breast cancer.  **Fig C. The flow chart and results of case-matched selection of BC-only patients**.

**
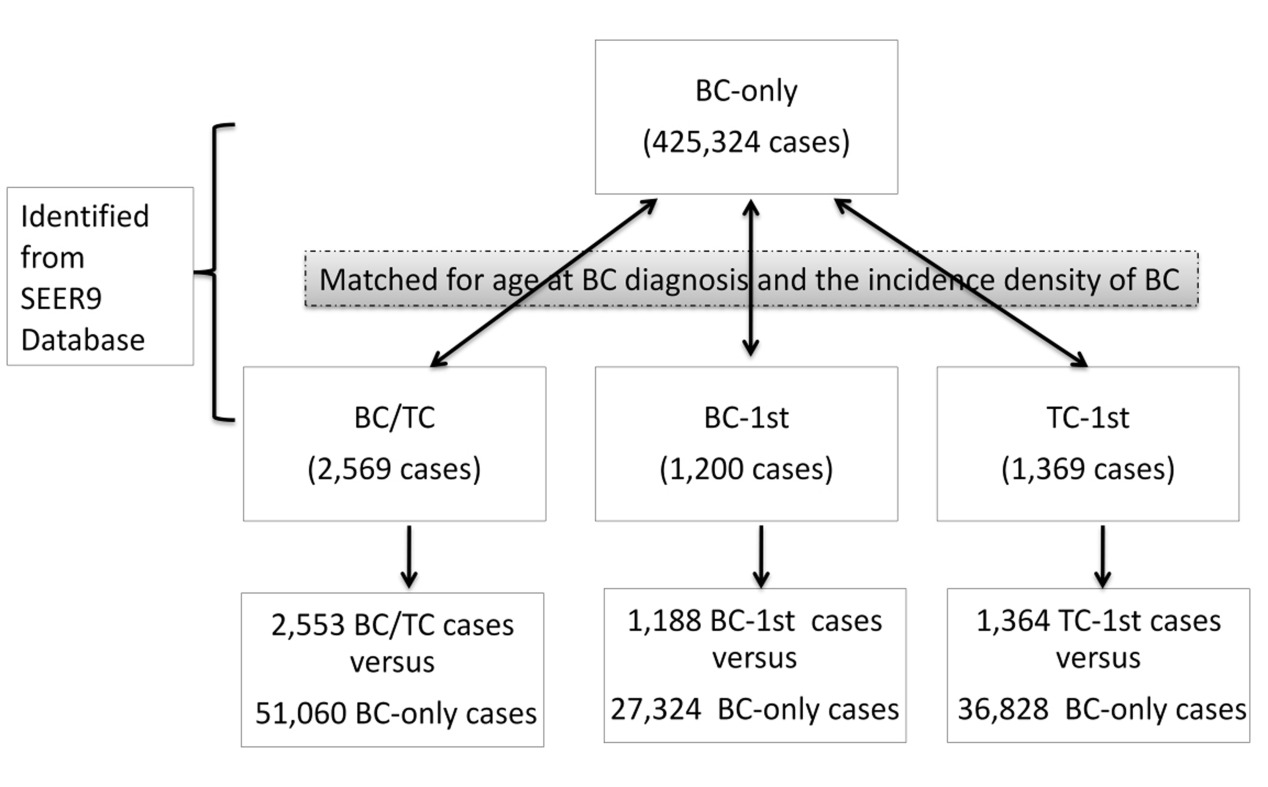
**

Illustrated is the flow chart of the case-matched selection of BC-only patients to match patients in the BC/TC, BC-1st, and TC-1st groups for patient age and incidence densities of BC in individual consecutive follow-up years. The number of cases indicated in each group at the final step was the actual number of cases that could be matched and identified, which were finally used for this study. **BC**, breast cancer; **TC**, thyroid cancer; **BC-only**, patients only with a diagnosis of breast cancer without a history of thyroid cancer; **BC/TC**, breast cancer patients also with a history of thyroid cancer diagnosed any time—either before or after the diagnosis of breast cancer; **BC-1st**, breast cancer was diagnosed first, followed by diagnosis of thyroid cancer; **TC-1st**, thyroid cancer was diagnosed first, followed by diagnosis of breast cancer.

**Fig D. Comparison of the cumulative mortalities of patients between matched BC-only patients and BC patients also with a history of TC**.


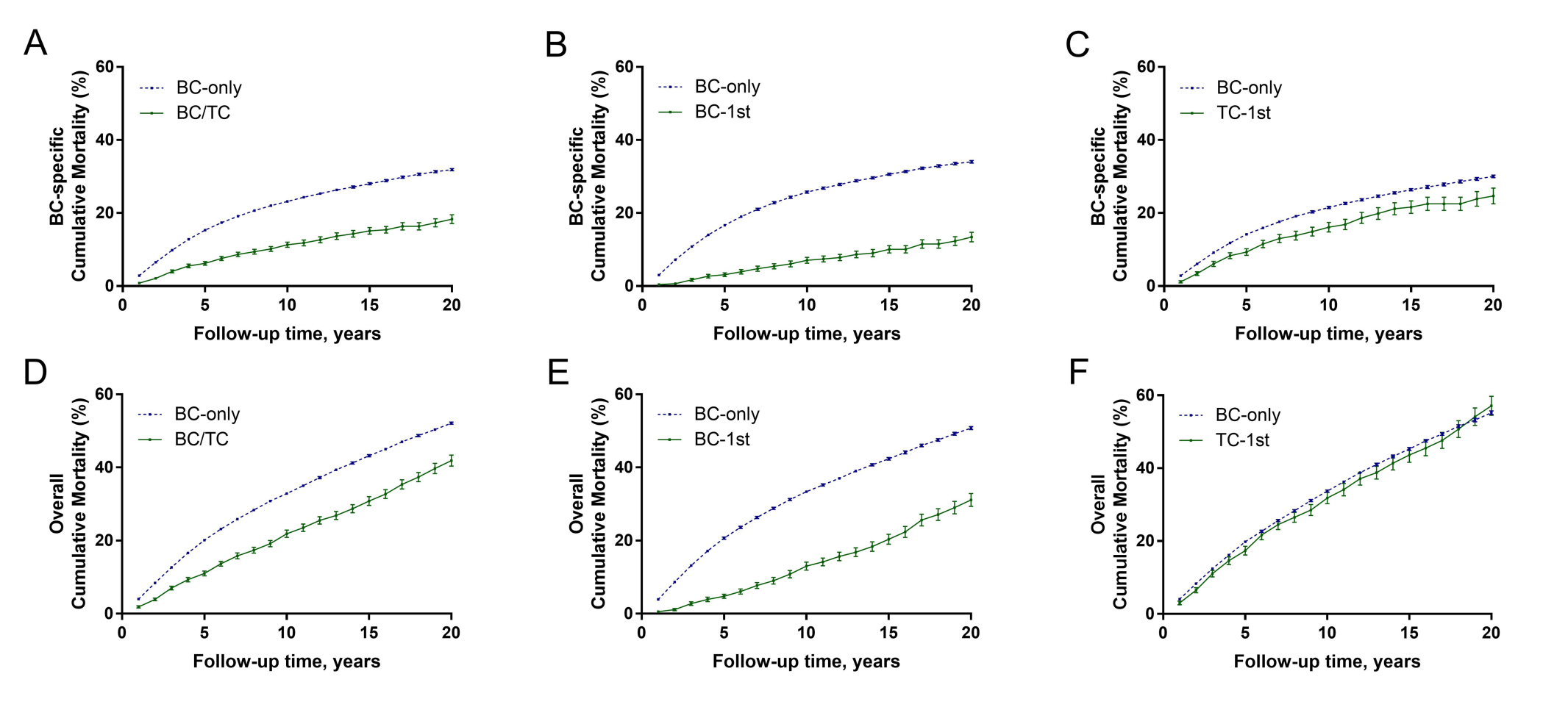


**A-C**, shown are BC-specific cumulative mortalities after the diagnosis of BC. **D-F**, shown are overall cumulative mortalities from any cause after the diagnosis with BC. **BC**, breast cancer; **TC**, thyroid cancer; **BC-only**, patients only with a diagnosis of breast cancer without a history of thyroid cancer; **BC/TC**, breast cancer patients also with a history of thyroid cancer diagnosed any time—either before or after the diagnosis of breast cancer; **BC-1st**, breast cancer was diagnosed first, followed by diagnosis of thyroid cancer; **TC-1st**, thyroid cancer was diagnosed first, followed by diagnosis of breast cancer.

**Fig E. Comparison of the cumulative mortalities of patients between matched BC-only patients and BC patients also with a history of TC in white people**

**
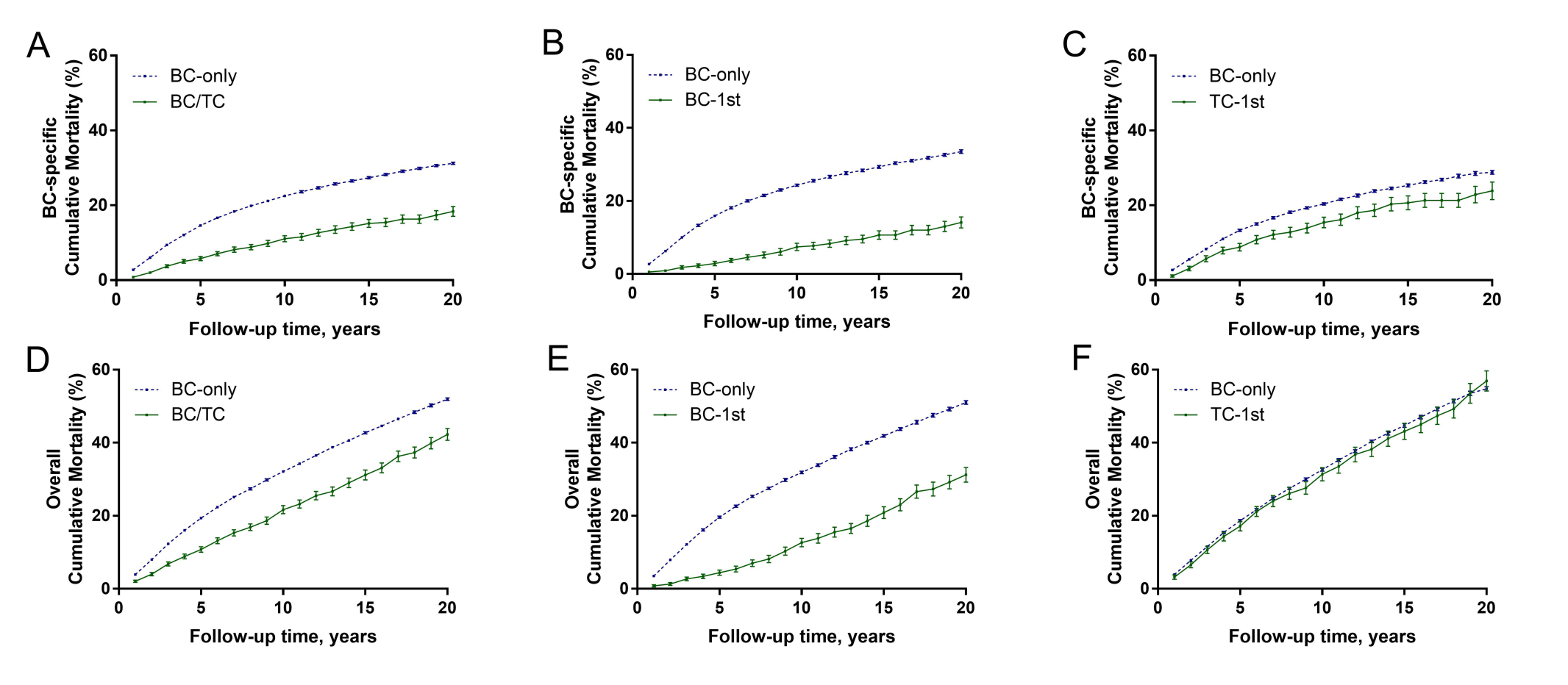
**

1. **C**, shown are BC-specific cumulative mortalities after the diagnosis of BC. **D-F**, shown are overall cumulative mortalities from any cause after the diagnosis with BC. **BC**, breast cancer; **TC**, thyroid cancer; **BC-only**, patients only with a diagnosis of breast cancer without a history of thyroid cancer; **BC/TC**, breast cancer patients also with a history of thyroid cancer diagnosed any time—either before or after the diagnosis of breast cancer; **BC-1st**, breast cancer was diagnosed first, followed by diagnosis of thyroid cancer; **TC-1st**, thyroid cancer was diagnosed first, followed by diagnosis of breast cancer.

**Fig F. Kaplan-Meier analysis of the effect of a history of TC on overall survivals of patients in BC/TC and TC-1st patients**


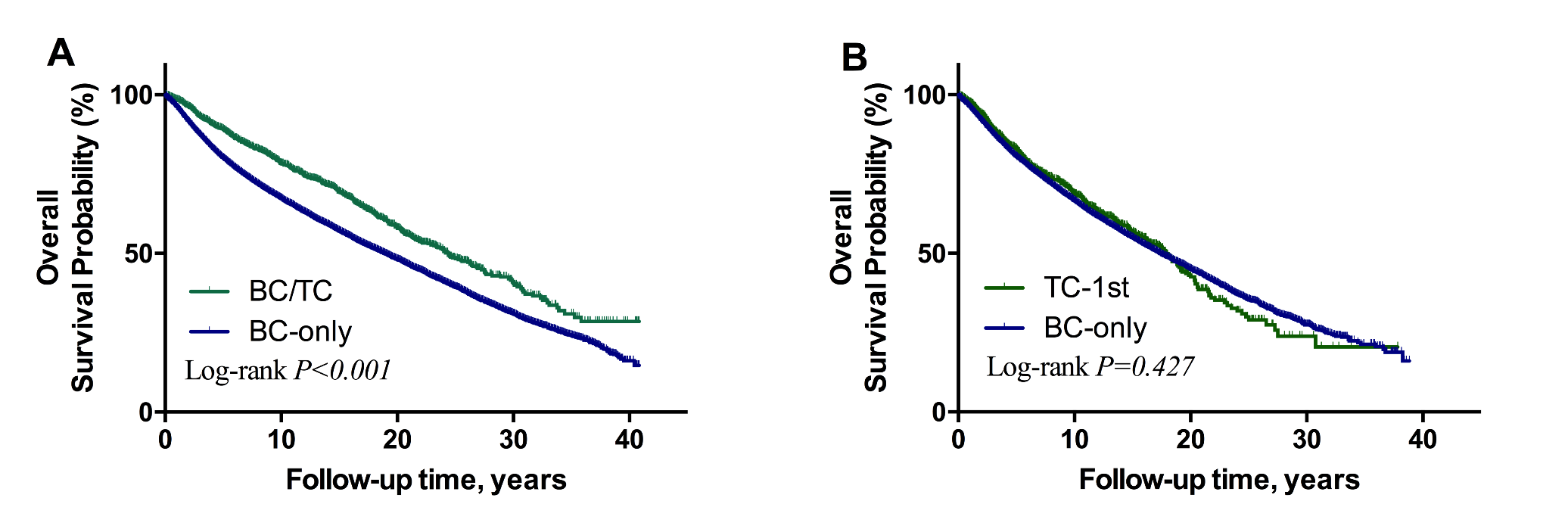


**A**, comparison of overall survival curves between BC/TC and matched BC-only patients. **B**, comparison of overall survival curves between TC-1st and matched BC-only patients. **BC**, breast cancer; **TC**, thyroid cancer; **BC-only**, patients only with a diagnosis of breast cancer without a history of thyroid cancer; **BC/TC**, breast cancer patients also with a history of thyroid cancer diagnosed any time—either before or after the diagnosis of breast cancer; **BC-1st**, breast cancer was diagnosed first, followed by diagnosis of thyroid cancer; **TC-1st**, thyroid cancer was diagnosed first, followed by diagnosis of breast cancer.

**Fig G. Kaplan-Meier analysis of the effect of a history of TC on BC-specific and overall survivals of white patients in various settings**.

**
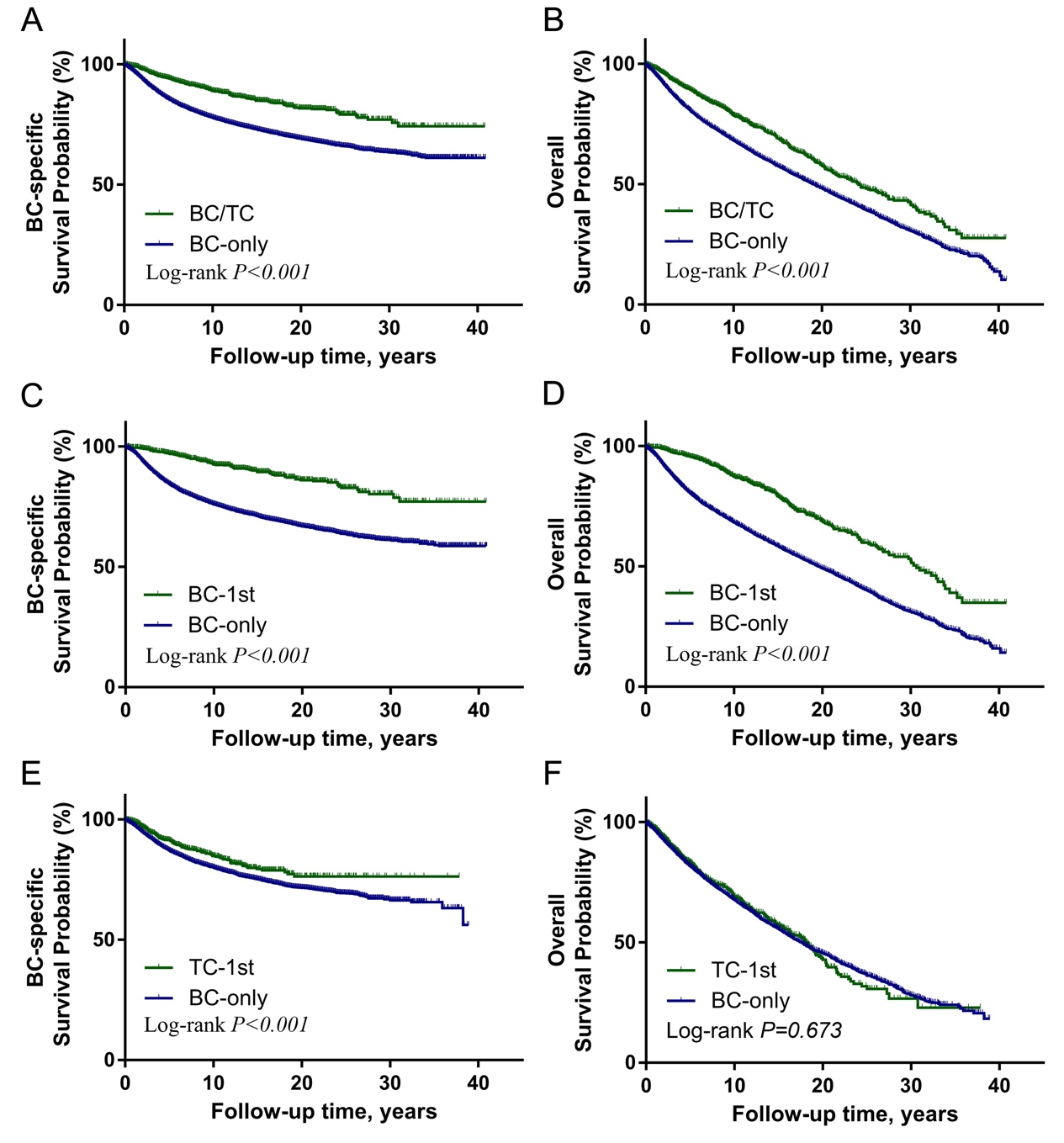
**

**A**, comparison of BC-specific survival curves between BC/TC and matched BC-only patients; **B**, comparison of overall survival curves between BC/TC and matched BC-only patients. **C**, comparison of BC-specific survival curves between BC-1st and matched BC-only patients; **D**, comparison of overall survival curves between BC-1st and matched BC-only patients. **E**, comparison of BC-specific survival curves between TC-1st and matched BC-only patients; **F**, comparison of overall survival curves between TC-1st and matched BC-only patients. **BC**, breast cancer; **TC**, thyroid cancer; **BC-only**, patients only with a diagnosis of breast cancer without a history of thyroid cancer; **BC/TC**, breast cancer patients also with a history of thyroid cancer diagnosed any time—either before or after the diagnosis of breast cancer; **BC-1st**, breast cancer was diagnosed first, followed by diagnosis of thyroid cancer; **TC-1st**, thyroid cancer was diagnosed first, followed by diagnosis of breast cancer.

**Fig H. Comparison of the cumulative mortalities of patients between BC patients with a history of TC and matched patients only with BC (data from the second random case-matched selection)**.

**
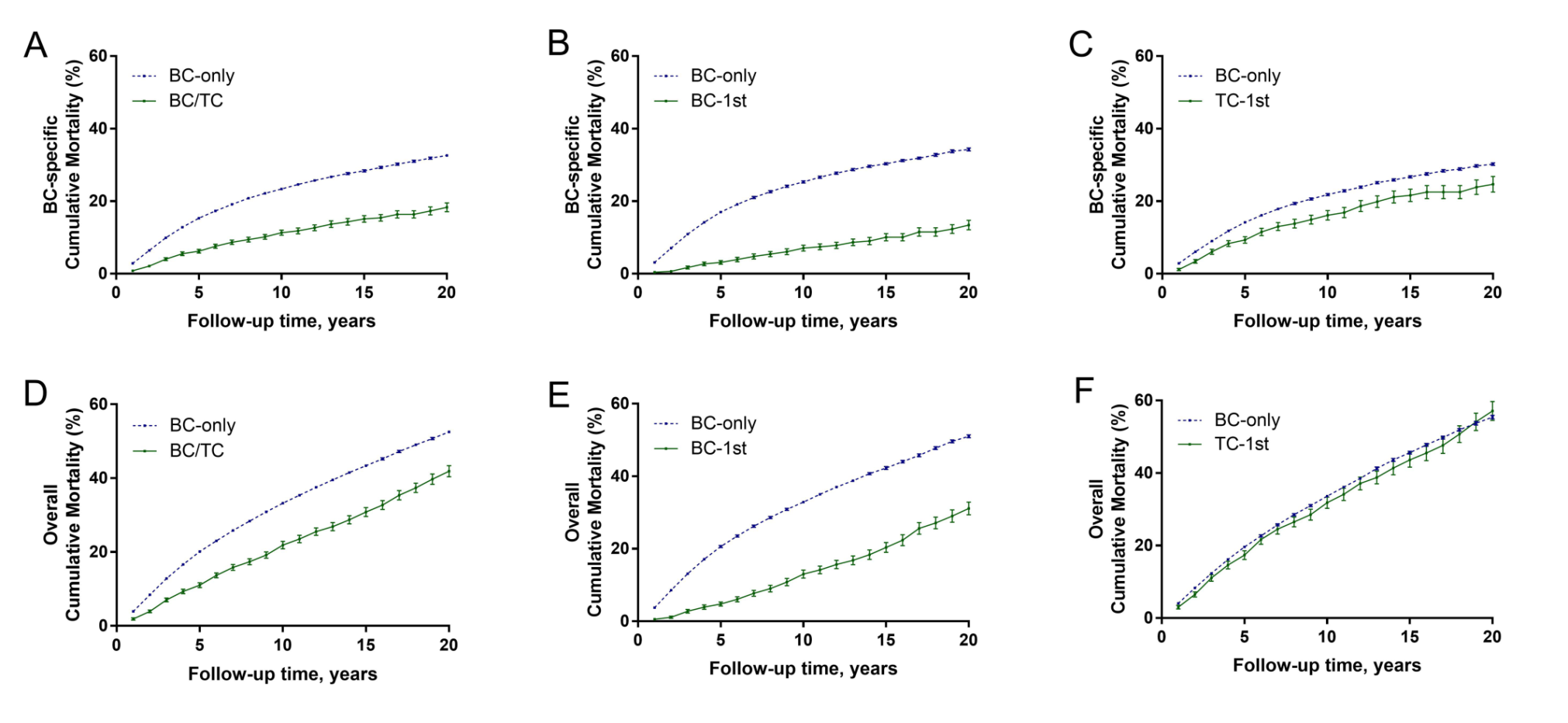
**

**A-C**, shown are BC-specific cumulative mortalities after the diagnosis of BC. **D-F**, shown are overall cumulative mortalities from any cause after the diagnosis with BC. **BC**, breast cancer; **TC**, thyroid cancer; **BC-only**, patients only with a diagnosis of breast cancer without a history of thyroid cancer; **BC/TC**, breast cancer patients also with a history of thyroid cancer diagnosed any time—either before or after the diagnosis of breast cancer; **BC-1st**, breast cancer was diagnosed first, followed by diagnosis of thyroid cancer; **TC-1st**, thyroid cancer was diagnosed first, followed by diagnosis of breast cancer.

**Fig I. Kaplan-Meier analysis of the effect of a history of TC on BC-specific and overall survivals of patients (data from the second random case-matched selection)**.


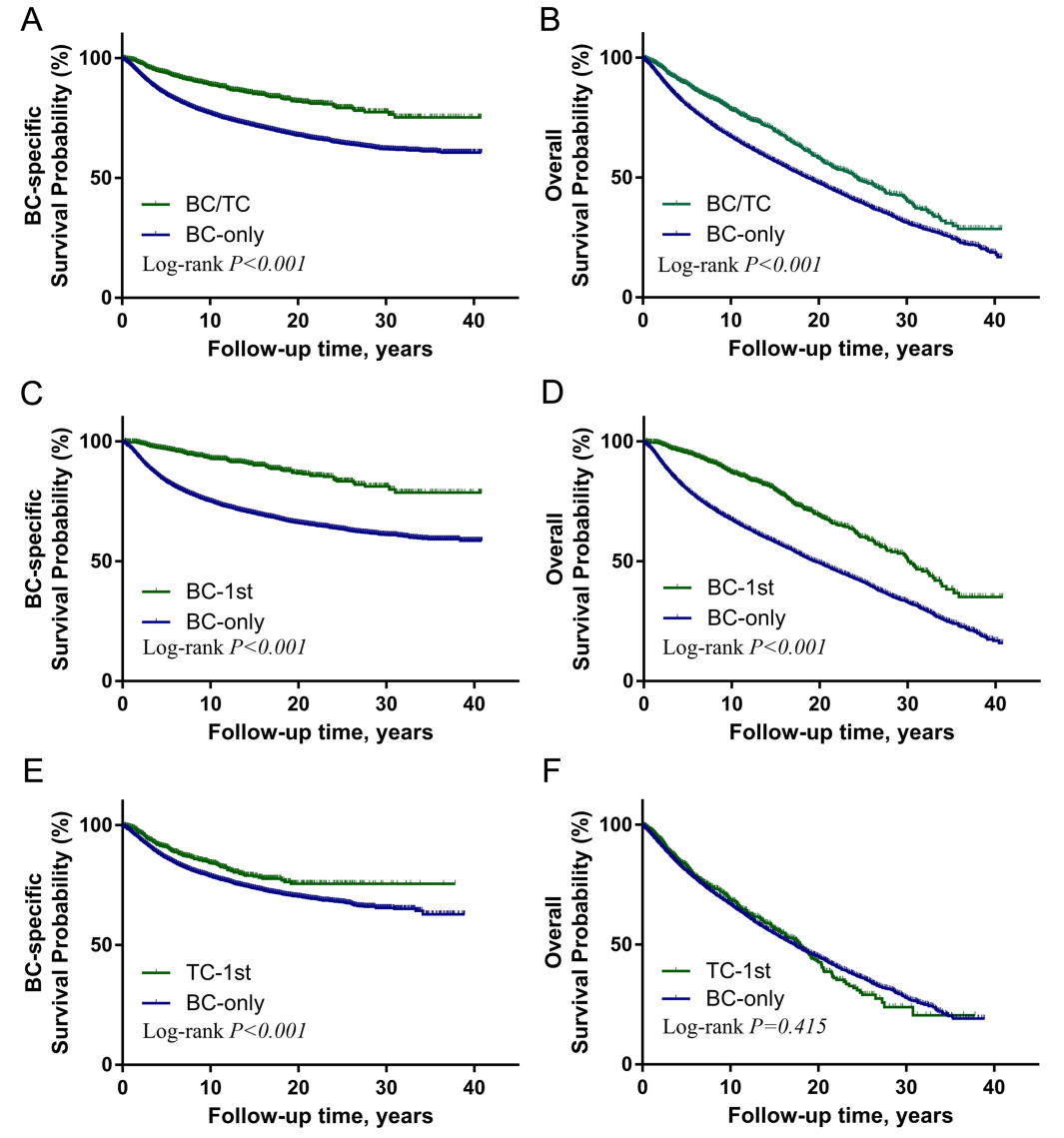


**A**, comparison of BC-specific survival curves between BC/TC and matched BC-only patients; **B**, comparison of overall survival curves between BC/TC and matched BC-only patients. **C**, comparison of BC-specific survival curves between BC-1st and matched BC-only patients; **D**, comparison of overall survival curves between BC-1st and matched BC-only patients. **E**, comparison of BC-specific survival curves between TC-1st and matched BC-only patients; **F**, comparison of overall survival curves between TC-1st and matched BC-only patients. **BC**, breast cancer; **TC**, thyroid cancer; **BC-only**, patients only with a diagnosis of breast cancer without a history of thyroid cancer; **BC/TC**, breast cancer patients also with a history of thyroid cancer diagnosed any time—either before or after the diagnosis of breast cancer; **BC-1st**, breast cancer was diagnosed first, followed by diagnosis of thyroid cancer; **TC-1st**, thyroid cancer was diagnosed first, followed by diagnosis of breast cancer.

**Fig J. Comparison of the cumulative mortalities of patients between BC patients with a history of TC and matched patients only with BC (data from the third random case-matched selection)**.


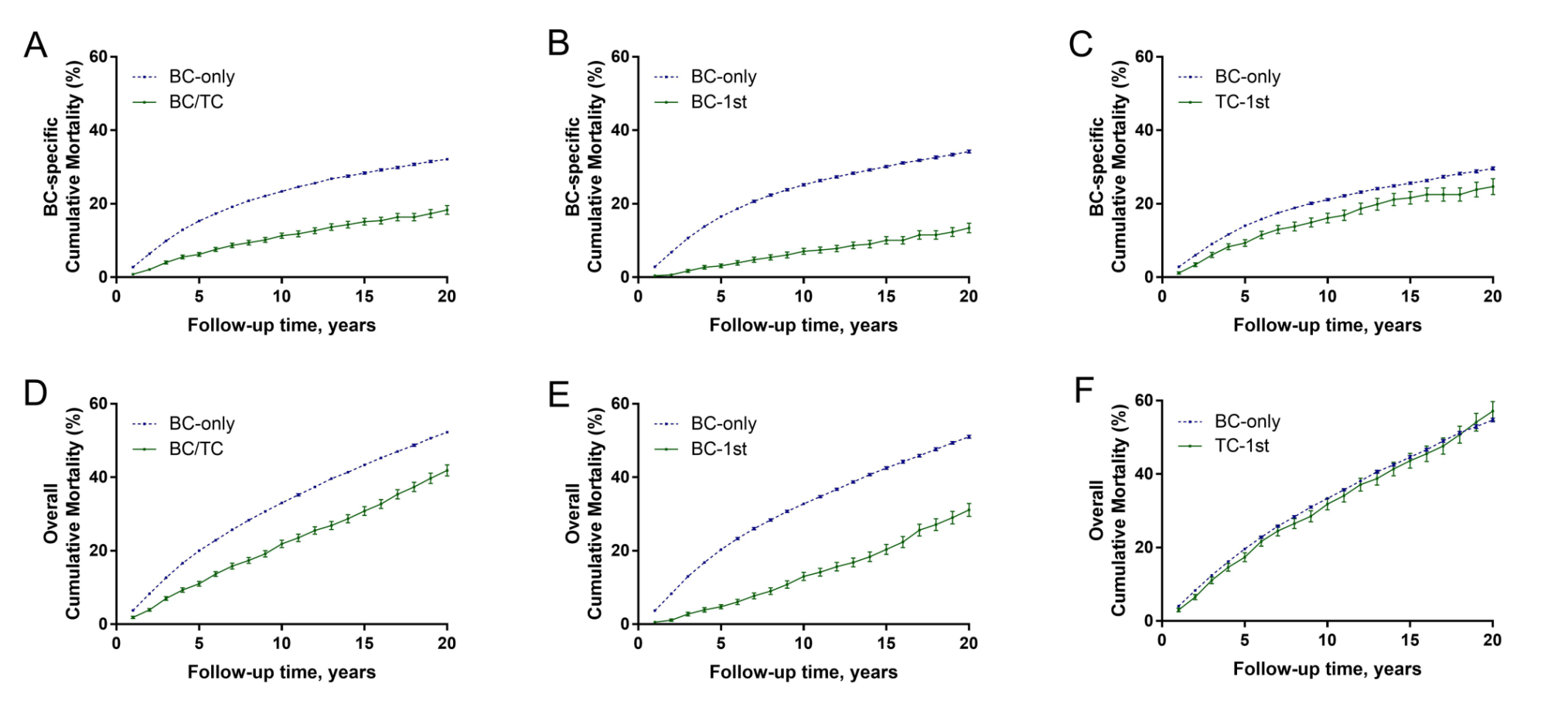


**A-C**, shown are BC-specific cumulative mortalities after the diagnosis of BC. **D-F**, shown are overall cumulative mortalities from any cause after the diagnosis with BC. **BC**, breast cancer; **TC**, thyroid cancer; **BC-only**, patients only with a diagnosis of breast cancer without a history of thyroid cancer; **BC/TC**, breast cancer patients also with a history of thyroid cancer diagnosed any time—either before or after the diagnosis of breast cancer; **BC-1st**, breast cancer was diagnosed first, followed by diagnosis of thyroid cancer; **TC-1st**, thyroid cancer was diagnosed first, followed by diagnosis of breast cancer.

**Fig K. Kaplan-Meier analysis of the effect of a history of TC on BC-specific and overall survivals of patients (data from the third random case-matched selection)**.


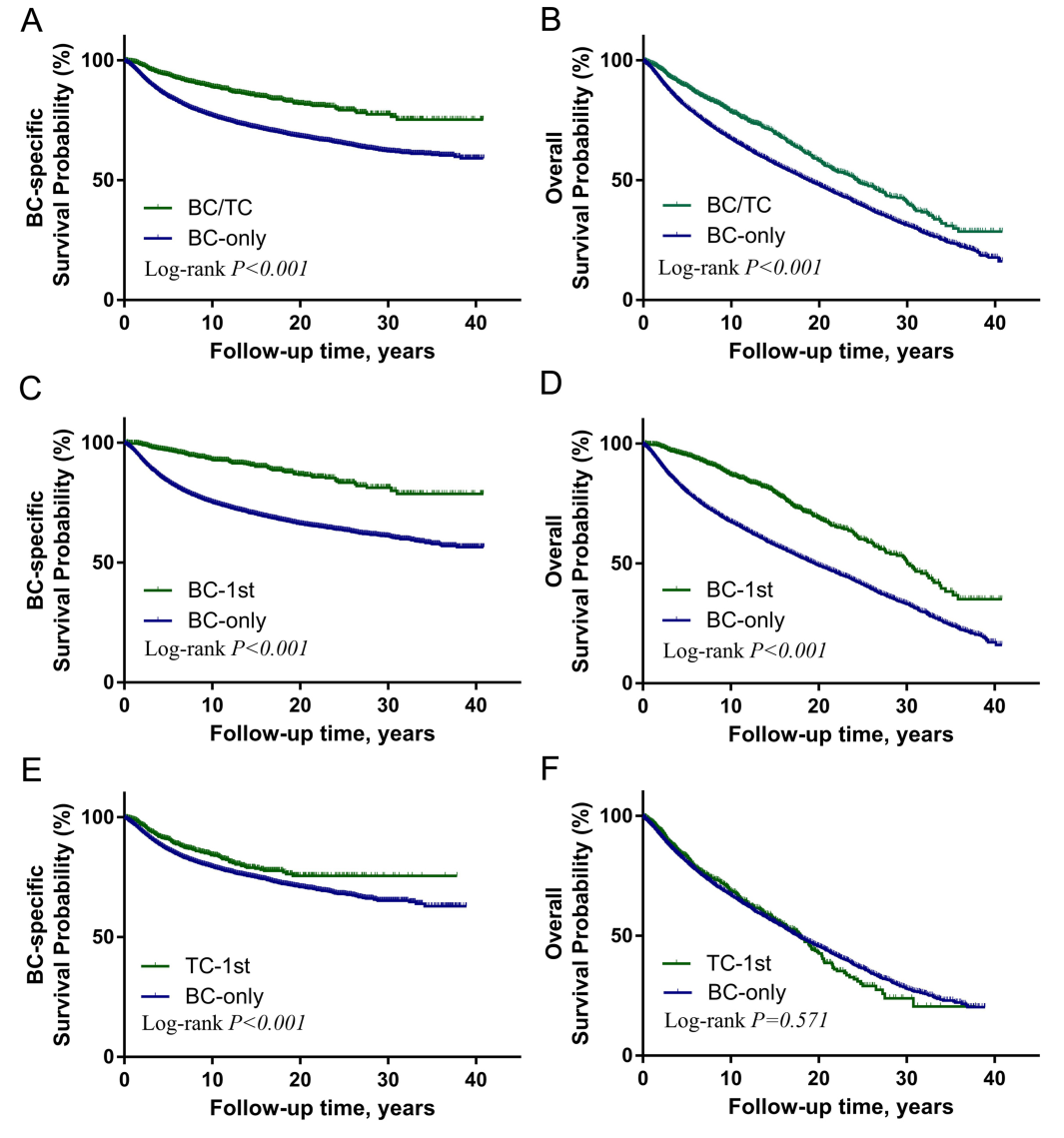


**A**, comparison of BC-specific survival curves between BC/TC and matched BC-only patients; **B**, comparison of overall survival curves between BC/TC and matched BC-only patients. **C**, comparison of BC-specific survival curves between BC-1st and matched BC-only patients; **D**, comparison of overall survival curves between BC-1st and matched BC-only patients. **E**, comparison of BC-specific survival curves between TC-1st and matched BC-only patients; **F**, comparison of overall survival curves between TC-1st and matched BC-only patients. **BC**, breast cancer; **TC**, thyroid cancer; **BC-only**, patients only with a diagnosis of breast cancer without a history of thyroid cancer; **BC/TC**, breast cancer patients also with a history of thyroid cancer diagnosed any time—either before or after the diagnosis of breast cancer; **BC-1st**, breast cancer was diagnosed first, followed by diagnosis of thyroid cancer; **TC-1st**, thyroid cancer was diagnosed first, followed by diagnosis of breast cancer.

**Fig L. Comparison of the cumulative mortalities between breast cancer patients with a history of papillary thyroid cancer and matched patients with breast cancer only**


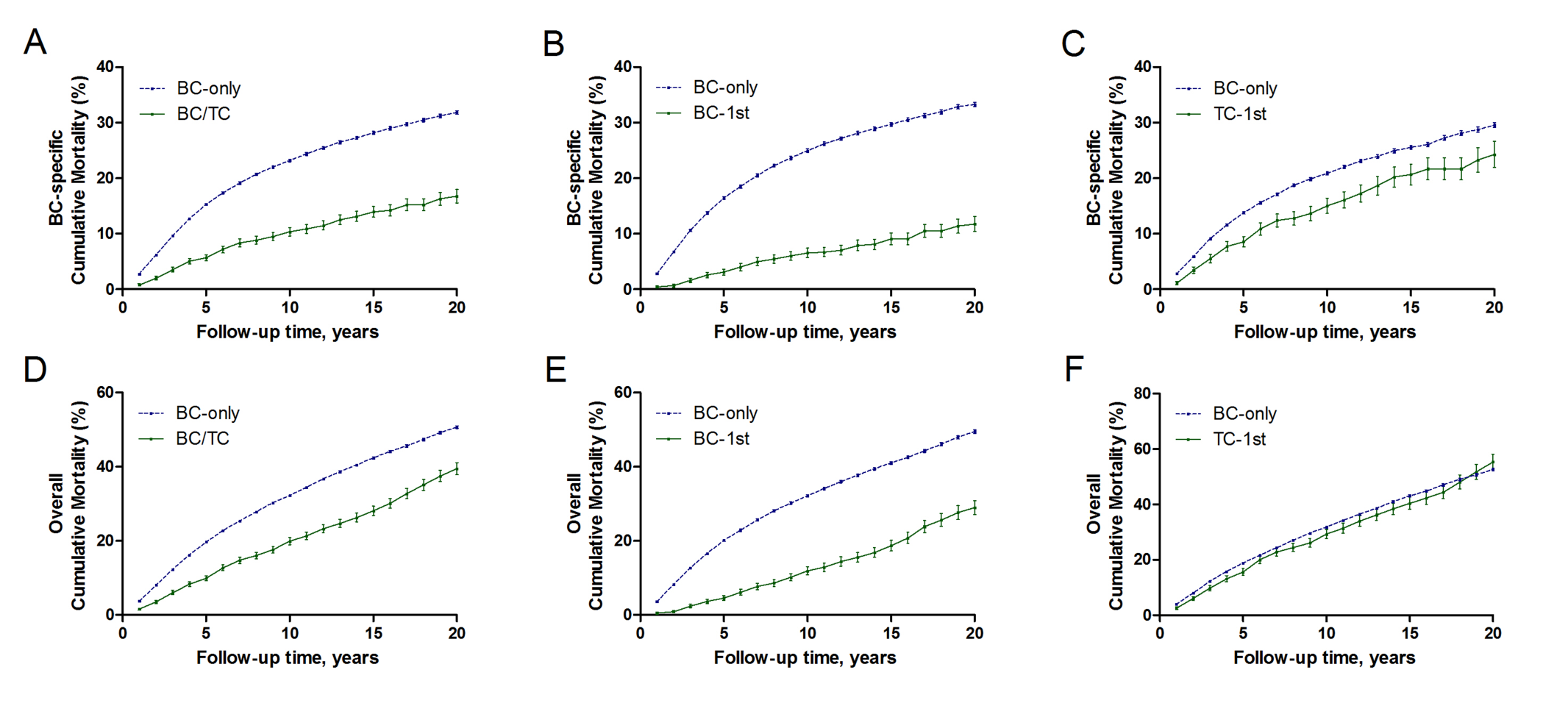


**A-C**, shown are BC-specific cumulative mortalities after the diagnosis of BC. **D-F**, shown are overall cumulative mortalities from any cause after the diagnosis with BC. **BC**, breast cancer; **TC**, thyroid cancer; **BC-only**, patients only with a diagnosis of breast cancer without a history of thyroid cancer; **BC/TC**, breast cancer patients also with a history of thyroid cancer diagnosed any time—either before or after the diagnosis of breast cancer; **BC-1st**, breast cancer was diagnosed first, followed by diagnosis of thyroid cancer; **TC-1st**, thyroid cancer was diagnosed first, followed by diagnosis of breast cancer.

**Fig M. Kaplan-Meier analysis of the effects of a history of papillary thyroid cancer on breast cancer-specific and overall survivals of patients in various settings**.


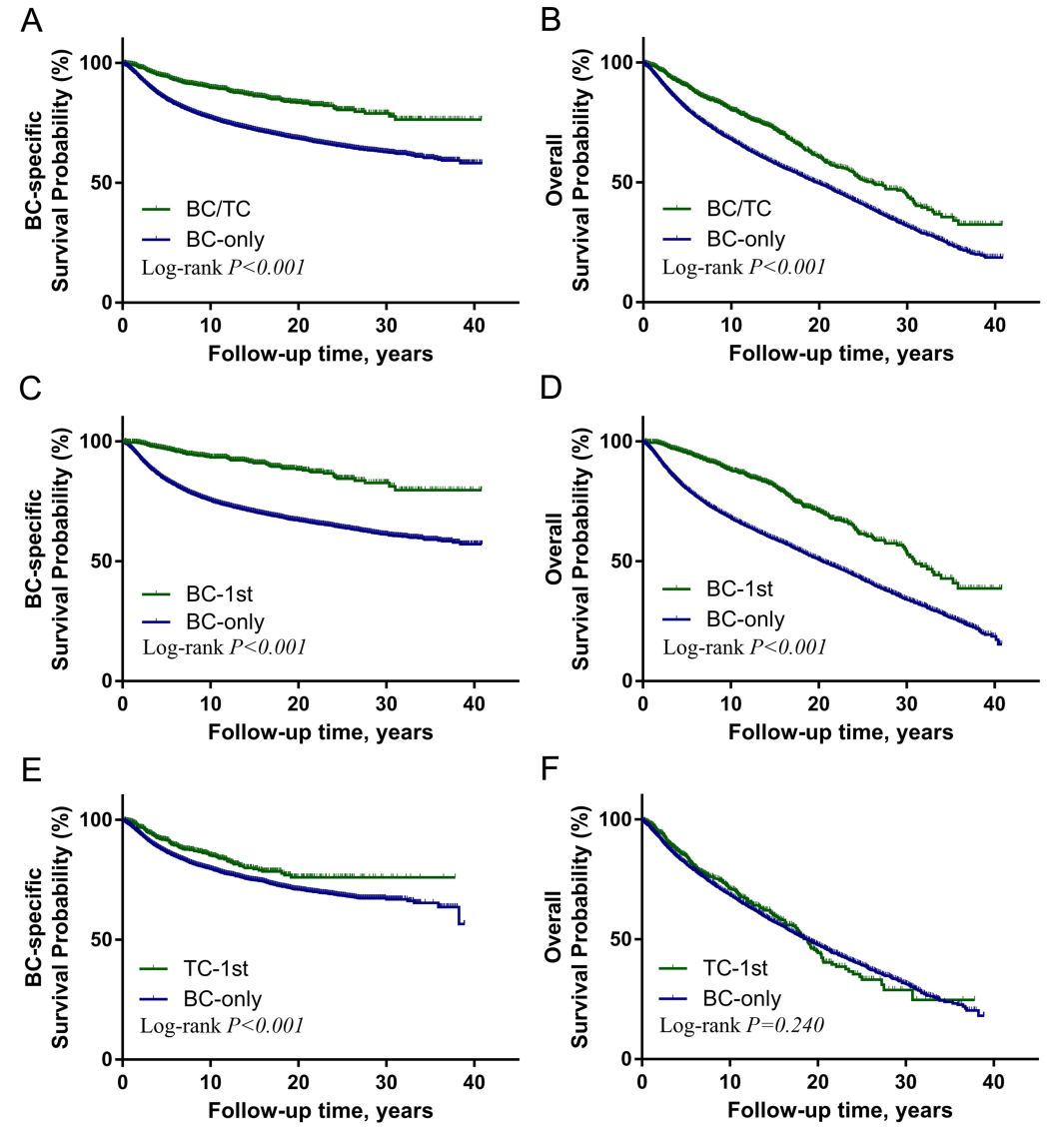


**A**, comparison of BC-specific survival curves between BC/TC and matched BC-only patients; **B**, comparison of overall survival curves between BC/TC and matched BC-only patients. **C**, comparison of BC-specific survival curves between BC-1st and matched BC-only patients; **D**, comparison of overall survival curves between BC-1st and matched BC-only patients. **E**, comparison of BC-specific survival curves between TC-1st and matched BC-only patients; **F**, comparison of overall survival curves between TC-1st and matched BC-only patients. **BC**, breast cancer; **TC**, papillary thyroid cancer; **BC-only**, patients only with a diagnosis of breast cancer without a history of thyroid cancer; **BC/TC**, breast cancer patients also with a history of papillary thyroid cancer diagnosed any time—either before or after the diagnosis of breast cancer; **BC-1st**, breast cancer was diagnosed first, followed by diagnosis of papillary thyroid cancer; **TC-1st**, papillary thyroid cancer was diagnosed first, followed by diagnosis of breast cancer.

**Fig N. Kaplan-Meier analyses of the differential protective effects of a history of papillary thyroid cancer on breast cancer-specific survival between younger and older patients**.


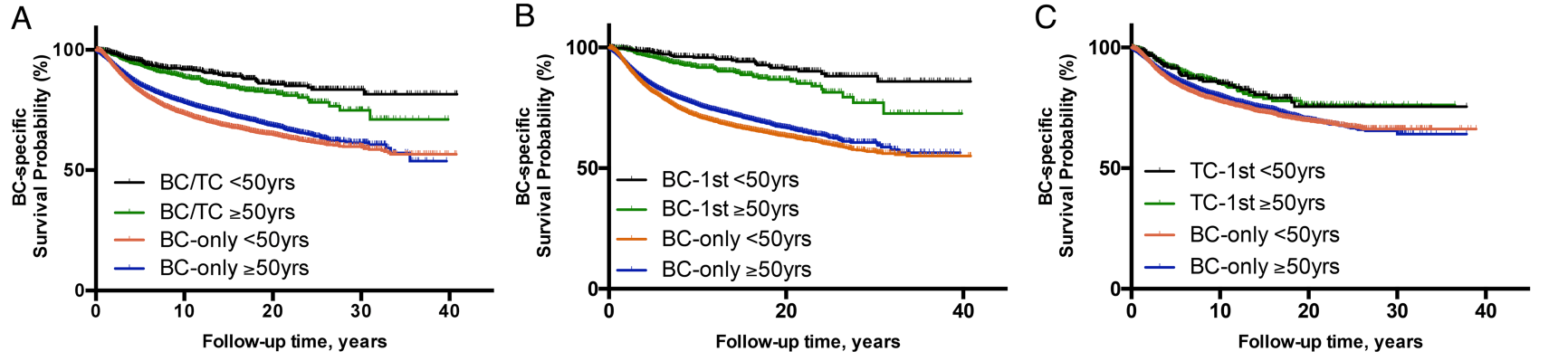


**A**, shown are the comparisons of the effects of a history of TC on BC-specific survival curves in BC/TC and matched BC-only patients between the group aged < 50 years and the group aged ≥ 50 years. **B**, shown are the comparisons of the effects of a history of TC on BC-specific survival curves in BC-1st and matched BC-only patients between the group aged < 50 years and the group aged ≥ 50 years. **C**, shown are the comparisons of the effects of a history of TC on BC-specific survival curves in TC-1st and matched BC-only patients between the group aged < 50 years and the group aged ≥ 50 years. **BC**, breast cancer; **TC**, papillary thyroid cancer; **BC-only**, patients only with a diagnosis of breast cancer without a history of thyroid cancer; **BC/TC**, breast cancer patients also with a history of papillary thyroid cancer diagnosed any time—either before or after the diagnosis of breast cancer; **BC-1st**, breast cancer was diagnosed first, followed by diagnosis of papillary thyroid cancer; **TC-1st**, papillary thyroid cancer was diagnosed first, followed by the diagnosis of breast cancer.

**Table A. Comparison of clinicopathological characteristics of breast cancer in various clinical settings in white people**

|  | **Comparison 1** | | | **Comparison 2** | | | **Comparison 3** | | |
| --- | --- | --- | --- | --- | --- | --- | --- | --- | --- |
|  | **BC/TC n/N(%)** | **BC-only n/N(%)** | **P value** | **BC-1st n/N(%)** | **BC-only n/N(%)** | **P value** | **TC-1st n/N(%)** | **BC-only n/N(%)** | **P value** |
| Number of cases | 1970 | 39400 |  | 907 | 18140 |  | 1067 | 23474 |  |
| Age at diagnosis (yrs)；  Median (IQR) | 57 (49-67) | 57 (49-67) | 1.000 | 54 (46-63) | 54 (46-63) | 1.000 | 61 (52-70) | 61 (52-70) | 1.000 |
| Tumor Size(mm)；  Median (IQR) | 15 (10-25) | 16 (10-25) | <0.001 | 15 (10-25) | 17 (11-26) | 0.001 | 15 (10-23) | 16 (10-25) | 0.001 |
| Pathology Ductal | 1558/1970  (79.1) | 31088/39400 (78.8) | 0.863 | 728/907 (80.3) | 14304/18140 (78.8) | 0.317 | 833/1067 (78.1) | 18340/23474 (78.1) | 0.972 |
| Lobular | 173/1970  (8.8) | 3146/39400 (8.0) | 0.201 | 75/907  (8.3) | 1409/18140 (7.8) | 0.572 | 98/1067 (9.2) | 2085/23474 (8.9) | 0.746 |
| Mixed | 170/1970  (8.6) | 3289/39400 (8.3) | 0.650 | 65/907  (7.2) | 1354/18140 (7.5) | 0.790 | 105/1067 (9.8) | 2148/23474 (9.2) | 0.450 |
| Inflammatory | 9/1970  (0.5) | 245/39400  (0.6) | 0.459 | 5/907  (0.6) | 144/18140  (0.8) | 0.561 | 4/1067  (0.4) | 111/23474  (0.5) | 0.820 |
| ER/PR status ER-positive | 1205/1449  (83.2) | 23297/29326 (79.4) | 0.001 | 487/595 (81.8) | 9231/11928 (77.4) | 0.011 | 718/854 (84.1) | 15606/19133 (81.6) | 0.066 |
| PR-positive | 1035/1426  (72.6) | 20188/29064 (69.5) | 0.013 | 424/583 (72.7) | 8028/11792 (68.1) | 0.021 | 611/843 (72.5) | 13399/18982 (70.6) | 0.247 |
| Both positive | 1010/1426  (70.8) | 19563/29043 (67.4) | 0.007 | 409/583 (70.2) | 7722/11792 (65.5) | 0.021 | 601/843 (71.3) | 13106/18974 (69.1) | 0.183 |
| LN metastasis | 545/1756  (31.0) | 12024/34750 (34.6) | 0.002 | 244/767 (31.8) | 5605/15080 (37.2) | 0.003 | 301/989 (30.4) | 7113/21595 (32.9) | 0.104 |
| Distant Metastasis | 52/1942  (2.7) | 2539/38716 (6.6) | <0.001 | 19/894  (2.1) | 1165/17761 (6.6) | <0.001 | 33/1052 (3.1) | 1452/23122 (6.3) | <0.001 |
| AJCC Stage I+II | 1369/1578  (86.8) | 26070/31717 (82.2) | <0.001 | 564/651 (86.6) | 10699/13207 (81.0) | <0.001 | 805/927 (86.8 ) | 16906/20365 (83.0) | 0.002 |
| III+IV | 209/1578  (13.2) | 5647/31717 (17.8) |  | 87/651 (13.4) | 2508/13207 (19.0) |  | 122/927 (13.2) | 3459/20365 (17.0) |  |
| Radiation Therapy | 937/1928  (48.6) | 19354/38486 (50.3) | 0.148 | 423/891 (47.5) | 8309/17717 (46.9) | 0.757 | 514/1041 (49.4) | 11924/22894 (52.1) | 0.093 |
| BC-specific Mortality | 224/1970  (11.4) | 8164/39400 (20.7) | <0.001 | 96/907 (10.6) | 4676/18140 (25.8) | <0.001 | 129/1067 (12.1) | 3872/23474 (16.5) | <0.001 |
| Follow Up Time； Median (IQR) | 107  (46-191) | 84  (36-161) | <0.001 | 160 (85-243) | 108  (52-192) | <0.001 | 68 (29-140) | 67  (27-136) | 0.299 |
| With Insurance | 593/598  (99.2) | 11857/11969  (99.1) | 0.977 | 163/164  (99.4) | 3186/3222  (98.9) | 0.822 | 430/434  (99.1) | 9489/9569  (99.2) | 1 |
| Married*The numbers of unemployed cases and cases with an education of least bachelor degree were obtained by calculation based on the unemployment rates and rates of education of at least bachelor degree and the corresponding total cases, respectively, in different counties | 1286/1904  (67.5) | 24527/37946  (64.6) | 0.010 | 618/879  (70.3) | 11830/17513  (67.5) | 0.095 | 672/1029  (65.3) | 14006/22524  (62.2) | 0.047 |
| Unemployed * | 85/1159  (7.3) | 1716/23167  (7.4) | 0.972 | 29/432  (6.7) | 587/8634  (6.8) | 1.000 | 56/727  (7.7) | 1240/15985  (7.8) | 1.000 |
| Education (at lease bachelor degree) * | 355/1159  (30.6) | 7149/23167  (30.9) | 0.895 | 128/432  (29.6) | 2563/8634  (29.7) | 1.000 | 227/727  (31.2) | 4991/15985  (31.2) | 1.000 |
| Median family income (US dollar); Median (IQR) | 65860  (50980-79000) | 65860  (51440-79410) | 0.792 | 60550  (46610-75540) | 60730  (47400-75540) | 0.785 | 66610  (52420-80800) | 66610  (53000-80860) | 0.804 |

**Notes**: Abbreviations: **BC**, breast cancer; **TC**, thyroid cancer; **BC-only**, patients only with breast cancer without a history of thyroid cancer;**BC/TC**, breast cancer patients also with a history of thyroid cancer diagnosed any time—either before or after the diagnosis of breast cancer; **BC-1st**, breast cancer was diagnosed first, followed by diagnosis of thyroid cancer; **TC-1st**, thyroid cancer was diagnosed first, followed by diagnosis of breast cancer; **IQR**, interquartile range; **ER**, estrogen receptor; **PR**, progesterone receptor; **LN**, lymph node. *The numbers of unemployed cases and cases with an education of least bachelor degree were obtained by calculation based on the unemployment rates and rates of education of at least bachelor degree and the corresponding total cases, respectively, in different counties

**Table B. Effects of a history of thyroid cancer on breast cancer-specific mortality—deaths per 1000 person-years and hazard ratios in white people**

|  |  | **BC-specific Mortality** | | **Deaths per 1000 Person-Years (95% CI）** | **Unadjusted** | | | **Adjustment^a^** | | **Adjustment^b^** | |
| --- | --- | --- | --- | --- | --- | --- | --- | --- | --- | --- | --- |
|  |  |  |  |  | **HR (95% CI)** | **P Value** | **HR (95% CI)** | | **P**  **Value** | **HR (95% CI)** | **P**  **Value** |
| Comparison 1 | BC-only | | 8164/39400 (20.7) | 22.8 (22.3-23.3) | 1.000 |  | 1.000 | |  | 1.000 |  |
|  | BC/TC | | 224/1970 (11.4) | 10.5 (9.2-12.0) | 0.49 (0.43-0.56) | <0.001 | 0.67 (0.55-0.82) | | <0.001 | 0.50 (0.38-0.68) | <0.001 |
| Comparison 2 | BC-only | | 4676/18140 (25.8) | 23.7 (23.0-24.4) | 1.000 |  | 1.000 | |  | 1.000 |  |
|  | BC-1st | | 96/907 (10.6) | 7.4 (6.0-9.0) | 0.34 (0.27-0.41) | <0.001 | 0.53 (0.38-0.72) | | <0.001 | 0.29 (0.16-0.50) | <0.001 |
| Comparison 3 | BC-only | | 3872/23474 (16.5) | 21.7 (21.0-22.4) | 1.000 |  | 1.000 | |  | 1.000 |  |
|  | TC-1st | | 129/1067 (12.1) | 15.5 (13.0-18.5) | 0.72 (0.61-0.86) | <0.001 | 0.81 (0.62-1.05) | | 0.111 | 0.67 (0.48-0.95) | 0.025 |

**Notes:** 1) Adjustment^a^ was made for race, tumor size, pathology type, ER/PR status, LN metastasis, radiation therapy, marital status and zip code. 2) Adjustment^b^ was made for race, tumor size, pathology type, ER/PR status, LN metastasis, radiation therapy, marital status, employment status, education level of at least bachelor degree, and median family income. 3) **BC**, breast cancer; **TC**, thyroid cancer; **BC-only**, patients only with breast cancer without a history of thyroid cancer; **BC/TC**, breast cancer patients also with a history of thyroid cancer diagnosed any time—either before or after the diagnosis of breast cancer; **BC-1st**, breast cancer was diagnosed first, followed by diagnosis of thyroid cancer; **TC-1st**, thyroid cancer was diagnosed first, followed by diagnosis of breast cancer; **CI**, confident interval; **HR**, hazard ratio; **ER**, estrogen receptor; **PR**, progesterone receptor; **LN**, lymph node.

**Table C. Comparison of clinicopathological characteristics of breast cancer in various clinical settings (data from the second case selection)**

|  | **BC/TC versus BC-only, n/N(%)** | | | **BC-1st versus BC-only, n/N(%)** | | | **TC-1st versus BC-only, n/N(%)** | | |
| --- | --- | --- | --- | --- | --- | --- | --- | --- | --- |
|  | **BC/TC** | **BC-only** | **P**  **value** | **BC-1st** | **BC-only** | **P**  **value** | **TC-1st** | **BC-only** | **P**  **value** |
| Number of cases | 2553 | 51060 |  | 1188 | 27324 |  | 1364 | 36828 |  |
| Age at diagnosis(yrs);  Median (IQR) | 57 (48-67) | 57 (48-67) | 1.000 | 54 (46-63) | 54 (46-63) | 1.000 | 60 (51-70) | 60 (51-70) | 1.000 |
| Tumor Size(mm);  median (IQR) | 16 (10-25) | 17 (10-27) | <0.001 | 16 (10-25) | 18 (11-28) | <0.001 | 15 (10-25) | 17 (10-27) | <0.001 |
| Pathology Ductal | 2044/2553 (80.1) | 40686/51060 (79.7) | 0.667 | 969/1188 (81.6) | 21898/27324 (80.1) | 0.235 | 1074/1364 (78.7) | 29115/36828 (79.1) | 0.789 |
| Lobular | 208/2553 (8.1) | 3792/51060 (7.4) | 0.176 | 86/1188 (7.2) | 1896/27324 (6.9) | 0.688 | 122/1364 (8.9) | 3080/36828 (8.4) | 0.457 |
| Mixed | 212/2553 (8.3) | 4130/51060 (8.1) | 0.686 | 83/1188 (7.0) | 1959/27324 (7.2) | 0.857 | 129/1364 (9.5) | 3217/36828 (8.7) | 0.355 |
| Inflammatory | 11/2553  (0.4) | 356/51060 (0.7) | 0.145 | 6/1188 (0.5) | 216/27324 (0.8) | 0.395 | 5/1364 (0.4) | 195/36828 (0.5) | 0.565 |
| ER/PR status ER-positive | 1551/1901 (81.6) | 30109/38548 (78.1) | <0.001 | 628/788 (79.7) | 13953/18376 (75.9) | 0.016 | 922/1112 (82.9) | 24277/30410 (79.8) | 0.013 |
| PR-positive | 1334/1877 (71.1) | 26008/38212 (68.1) | 0.006 | 548/776 (70.6) | 12179/18167 (67.0) | 0.039 | 785/1100 (71.4) | 20822/30186 (69.0) | 0.098 |
| Both positive | 1299/1876 (69.2) | 25188/38185 (66.0) | 0.004 | 528/776 (68.0) | 11691/18151 (64.4) | 0.039 | 770/1099 (70.1) | 20296/30171 (67.3) | 0.054 |
| LN metastasis | 737/2277 (32.4) | 16122/45131 (35.7) | 0.001 | 333/1009 (33.0) | 8873/22860 (38.8) | <0.001 | 404/1267 (31.9) | 11623/34087 (34.1) | 0.104 |
| Distant Metastasis | 82/2516  (3.3) | 3478/50126 (6.9) | <0.001 | 36/1168 (3.1) | 1841/26755 (6.9) | <0.001 | 46/1347 (3.4) | 2497/36252 (6.9) | <0.001 |
| AJCC Stage I+II | 1762/2059 (85.6) | 33693/41503 (81.2) | <0.001 | 734/862 (85.2) | 16164/20299 (79.6) | <0.001 | 1027/1196 (85.9) | 26321/32163 (81.8) | <0.001 |
| III+IV | 297/2059 (14.4) | 7810/41503 (18.8) |  | 128/862 (14.8) | 4135/20299 (20.4) |  | 169/1196 (14.1) | 5842/32163 (18.2) |  |
| Radiation Therapy | 1211/2498 (48.5) | 24957/49741 (50.2) | 0.101 | 555/1167 (47.6) | 12730/26641 (47.8) | 0.881 | 656/1330 (49.3) | 18964/35817 (52.9) | 0.010 |
| BC-specific mortality | 288/2553 (11.3) | 10859/51060 (21.3) | <0.001 | 118/1188 (9.9) | 7211/27324 (26.4) | <0.001 | 169/1364 (12.4) | 6286/36828 (17.1) | <0.001 |
| Follow Up Time;  median (IQR) | 102  (44-188) | 81  (35-156) | <0.001 | 155 (82-240) | 103  (49-188) | <0.001 | 65  (29-135) | 64  (26-130) | 0.150 |
| With Insurance | 801/809  (99.0) | 16023/16209  (98.9) | 0.806 | 225/229  (98.3) | 5177/5243  (98.7) | 0.732 | 575/579  (99.3) | 15475/15675  (98.7) | 0.293 |
| Married | 1629/2455  (66.4) | 30469/49065  (62.1) | <0.001 | 784/1142  (68.7) | 17182/26433  (65.0) | 0.0123 | 845/1312  (64.4) | 20999/35267  (59.5) | <0.001 |
| Unemployed * | 115/1513  (7.6) | 2336/30244  (7.7) | 0.900 | 40/575  (7.0) | 941/13212  (7.1) | 0.945 | 74/937  (7.9) | 2048/25285  (8.1) | 0.872 |
| Education (at lease bachelor degree) * | 467/1513  (30.9) | 9451/30244  (31.2) | 0.775 | 173/575  (30.1) | 3999/13212  (30.3) | 0.963 | 294/937  (31.4) | 8048/25285  (31.8) | 0.798 |
| Median family income (US dollar) ; Median (IQR) | 65540  (51440-80740) | 65860  (51650-80620) | 0.850 | 60550  (46610-79600) | 60730  (48010-75660) | 0.618 | 66610  (53000-82690) | 66610  (53000-81810) | 0.996 |

**Notes**: Abbreviations: **BC**, breast cancer; **TC**, thyroid cancer; **BC-only**, patients only with breast cancer without a history of thyroid cancer; **BC/TC**, breast cancer patients also with a history of thyroid cancer diagnosed any time—either before or after the diagnosis of breast cancer; **BC-1st**, breast cancer was diagnosed first, followed by diagnosis of thyroid cancer; **TC-1st**, thyroid cancer was diagnosed first, followed by diagnosis of breast cancer; **IQR**, interquartile range; **ER**, estrogen receptor; **PR**, progesterone receptor; **LN**, lymph node. *The numbers of unemployed cases and cases with an education of least bachelor degree were obtained by calculation based on the unemployment rates and rates of education of at least bachelor degree and the corresponding total cases, respectively, in different counties

**Table D. Effects of a history of thyroid cancer on breast cancer-specific mortality—deaths per 1000 person-years and hazard ratios (data from the second case selection)**

|  |  | **BC-specific Mortality** | **Deaths per 1000 Person-Years (95% CI）** | | **Unadjusted** | | | | | **Adjustment^a^** | | **Adjustment^b^** | |
| --- | --- | --- | --- | --- | --- | --- | --- | --- | --- | --- | --- | --- | --- |
|  |  |  |  |  | **HR (95% CI)** | | **P Value** | | | **HR (95% CI)** | **P Value** | **HR (95% CI)** | **P Value** |
| Comparison 1 | BC-only | 10859/51060 (21.3) | | 24.1 (23.6-24.5) | | 1.000 | |  | 1.000 | |  | 1.000 |  |
|  | BC/TC | 288/2553 (11.3) | | 10.7 (9.5-12.0) | | 0.47 (0.42-0.53) | | <0.001 | 0.66 (0.55-0.78) | | <0.001 | 0.54 (0.42-0.68) | <0.001 |
| Comparison 2 | BC-only | 7211/27324 (26.4) | | 24.8 (24.3-25.4) | | 1.000 | |  | 1.000 | |  | 1.000 |  |
|  | BC-1st | 118/1188 (9.9) | | 7.1 (5.8-8.5) | | 0.31 (0.26-0.37) | | <0.001 | 0.46 (0.34-0.61) | | <0.001 | 0.31(0.19-0.48) | <0.001 |
| Comparison 3 | BC-only | 6286/36828 (17.1) | | 23.5 (22.9-24.1) | | 1.000 | |  | 1.000 | |  | 1.000 |  |
|  | TC-1st | 169/1364 (12.4) | | 16.6 (14.2-19.3) | | 0.71 (0.61-0.83) | | <0.001 | 0.84 (0.67-1.05) | | 0.121 | 0.75 (0.56-1.00) | 0.048 |

**Notes:** 1) Adjustment^a^ was made for race, tumor size, pathology type, ER/PR status, LN metastasis, radiation therapy, marital status and zip code. 2) Adjustment^b^ was made for race, tumor size, pathology type, ER/PR status, LN metastasis, radiation therapy, married status, employment status, education of at least bachelor degree, and median family income. 3) **BC**, breast cancer; **TC**, thyroid cancer; **BC-only**, patients only with breast cancer without a history of thyroid cancer; **BC/TC**, breast cancer patients also with a history of thyroid cancer diagnosed any time—either before or after the diagnosis of breast cancer; **BC-1st**, breast cancer was diagnosed first, followed by diagnosis of thyroid cancer; **TC-1st**, thyroid cancer was diagnosed first, followed by diagnosis of breast cancer; **CI**, confident interval; **HR**, hazard ratio; **ER**, estrogen receptor; **PR**, progesterone receptor; **LN**, lymph node

**Table E. Comparison of clinicopathological characteristics of breast cancer in various clinical settings (data from the third case-matched selection)**

|  | **BC/TC versus BC-only, n/N(%)** | | | **BC-1st versus BC-only, n/N(%)** | | | **TC-1st versus BC-only, n/N(%)** | | |
| --- | --- | --- | --- | --- | --- | --- | --- | --- | --- |
|  | **BC/TC** | **BC-only** | **P value** | **BC-1st** | **BC-only** | **P value** | **TC-1st** | **BC-only** | **P value** |
| Number of cases | 2553 | 51060 |  | 1188 | 27324 |  | 1364 | 36828 |  |
| Age at diagnosis(yrs)；  Median (IQR) | 57 (48-67) | 57 (48-67) | 1.000 | 54 (46-63) | 54 (46-63) | 1.000 | 60 (51-70) | 60 (51-70) | 1.000 |
| Tumor Size(mm)；  Median (IQR) | 16 (10-25) | 17 (11-27) | <0.001 | 16 (10-25) | 18 (11-28) | <0.001 | 15 (10-25) | 17 (10-27) | <0.001 |
| Pathology Ductal | 2044/2553 (80.1) | 40834/51060 (80.0) | 0.938 | 969/1188 (81.6) | 21859/26643 (80.0) | 0.673 | 1074/1364 (78.7) | 29221/36828 (79.3) | 0.588 |
| Lobular | 208/2553 (8.1) | 3738/51060 (7.3) | 0.119 | 86/1188  (7.2) | 1987/26643 (7.3) | 0.816 | 122/1364  (8.9) | 3026/36828 (8.2) | 0.341 |
| Mixed | 212/2553 (8.3) | 4061/51060 (8.0) | 0.526 | 83/1188  (7.0) | 1958/26643 (7.2) | 0.686 | 129/1364  (9.5) | 3113/36828 (8.5) | 0.197 |
| Inflammatory | 11/2553 (0.4) | 321/51060 (0.6) | 0.245 | 6/1188  (0.5) | 193/26643  (0.7) | 0.482 | 5/1364  (0.4) | 239/36828  (0.6) | 0.294 |
| ER/PR status ER-positive | 1551/1901 (81.6) | 30141/38594 (78.1) | <0.001 | 628/788 (79.7) | 13902/18354 (75.7) | 0.011 | 922/1112 (82.9) | 24318/30577 (79.5) | 0.006 |
| PR-positive | 1334/1877 (71.1) | 26045/38276 (68.0) | 0.006 | 548/776 (70.6) | 12073/18146 (66.5) | 0.018 | 785/1100 (71.4) | 20847/30357 (68.7) | 0.060 |
| Both positive | 1299/1876 (69.2) | 25266/38255 (66.0) | 0.004 | 528/776 (68.0) | 11588/18131 (63.9) | 0.020 | 770/1099 (70.1) | 20282/30341 (66.8) | 0.027 |
| LN metastasis | 737/2277 (32.4) | 16257/45143 (36.0) | <0.001 | 333/1009 (33.0) | 8738/22825 (38.3) | 0.001 | 404/1267 (31.9) | 11686/34151 (34.2) | 0.092 |
| Distant Metastasis | 82/2516 (3.3) | 3351/50145 (6.7) | <0.001 | 36/1168  (3.1) | 1838/26740 (6.9) | <0.001 | 46/1347  (3.4) | 2430/36234 (6.7) | <0.001 |
| AJCC Stage I+II | 1762/2059 (85.6) | 33689/41467 (81.2) |  | 734/862 (85.2) | 16252/20248 (80.3) |  | 1027/1196 (85.9) | 26500/32187 (82.3) |  |
| III+IV | 297/2059 (14.4) | 7778/41467 (18.8) | <0.001 | 128/862 (14.8) | 3996/20248 (19.7) | <0.001 | 169/1196 (14.1) | 5687/32187 (17.7) | 0.002 |
| Radiation Therapy | 1211/2498 (48.5) | 24885/49715 (50.0) | 0.129 | 555/1167 (47.6) | 12789/26643 (48.0) | 0.788 | 656/1330 (49.3) | 18921/35799 (52.9) | 0.012 |
| BC-specific mortality | 288/2553 (11.3) | 10815/51060 (21.2) | <0.001 | 118/1188 (9.9) | 7141/27324 (26.1) | <0.001 | 169/1364 (12.4) | 6140/36828 (16.7) | <0.001 |
| Follow Up Time；  Median (IQR) | 102  (44-188) | 81  (35-156) | <0.001 | 155  (82-240) | 103  (50-187) | <0.001 | 65  (29-135) | 64  (26-131) | 0.155 |
| With Insurance | 801/809  (99.0) | 16027/16204  (98.9) | 0.918 | 225/229  (98.3) | 5149/5228  (98.5) | 0.542 | 575/579  (99.3) | 15513/15698  (98.8) | 0.380 |
| Married | 1629/2455  (66.4) | 30430/49056  (62.0) | <0.001 | 784/1142  (68.7) | 17040/26417  (64.5) | 0.005 | 845/1312  (64.4) | 20977/35288  (59.4) | <0.001 |
| Unemployed * | 115/1513  (7.6) | 2342/30239  (7.7) | 0.876 | 40/575  (7.0) | 939/13214  (7.1) | 0.957 | 74/937  (7.9) | 2045/25286  (8.1) | 0.882 |
| Education (at lease bachelor degree) * | 467/1513  (30.9) | 9446/30239  (31.2) | 0.782 | 173/575  (30.1) | 3998/13214  (30.3) | 0.968 | 294/937  (31.4) | 8060/25286  (31.9) | 0.775 |
| Median family income (US dollar); Median (IQR) | 65540  (51440-80740) | 65860  (51650-80620) | 0.917 | 60550  (46610-79600) | 60730  (48010-75540) | 0.699 | 66610  (53000-82690) | 66610  (53000-81810) | 0.975 |

**Notes**: Abbreviations: **BC**, breast cancer; **TC**, thyroid cancer; **BC-only**, patients only with breast cancer without a history of thyroid cancer; **BC/TC**, breast cancer patients also with a history of thyroid cancer diagnosed any time—either before or after the diagnosis of breast cancer; **BC-1st**, breast cancer was diagnosed first, followed by diagnosis of thyroid cancer; **TC-1st**, thyroid cancer was diagnosed first, followed by diagnosis of breast cancer; **IQR**, interquartile range; **ER**, estrogen receptor; **PR**, progesterone receptor; **LN**, lymph node. *The numbers of unemployed cases and cases with an education of least bachelor degree were obtained by calculation based on the unemployment rates and rates of education of at least bachelor degree and the corresponding total cases, respectively, in different counties

**Table F. Effects of a history of thyroid cancer on breast cancer-specific mortality—deaths per 1000 person-years and hazard ratios (data from the third case-matched selection)**

|  |  | **BC-specific Mortality** | **Deaths per 1000 Person-Years (95% CI）** | **Unadjusted** | | **Adjustment^a^** | | | **Adjustment^b^** | |
| --- | --- | --- | --- | --- | --- | --- | --- | --- | --- | --- |
|  |  |  |  | **HR (95% CI)** | **P Value** | **HR (95% CI)** | **P Value** | **HR (95% CI)** | | **P Value** |
| Comparison 1 | BC-only | 10815/51060 (21.2) | 23.9 (23.5-24.3) | 1.000 |  | 1.000 |  | 1.000 | |  |
|  | BC/TC | 288/2553 (11.3) | 10.7(9.5-12.0) | 0.47 (0.42-0.53) | <0.001 | 0.66 (0.56-0.79) | <0.001 | 0.55 (0.43-0.70) | | <0.001 |
| Comparison 2 | BC-only | 7141/27324 (26.1) | 24.7 (24.1-25.3) | 1.000 |  | 1.000 |  | 1.000 | |  |
|  | BC-1st | 118/1188 (9.9) | 7.1 (5.8-8.5) | 0.31 (0.26-0.37) | <0.001 | 0.47 (0.35-0.63) | <0.001 | 0.28 (0.17-0.44) | | <0.001 |
| Comparison 3 | BC-only | 6140/36828 (16.7) | 22.9 (22.3-23.5) | 1.000 |  | 1.000 |  | 1.000 | |  |
|  | TC-1st | 169/1364 (12.4) | 16.6 (14.2-19.3) | 0.73 (0.62-0.85) | <0.001 | 0.88 (0.71-1.11) | 0.283 | 0.76 (0.57-1.00) | | 0.053 |

**Notes:** 1) Adjustment^a^ was made for race, tumor size, pathology type, ER/PR status, LN metastasis, radiation therapy, marital status and zip code. 2) Adjustment^b^ was made for race, tumor size, pathology type, ER/PR status, LN metastasis, radiation therapy, married status, employment status, education of at least bachelor degree, and median family income. 3) **BC**, breast cancer; **TC**, thyroid cancer; **BC-only**, patients only with breast cancer without a history of thyroid cancer; **BC/TC**, breast cancer patients also with a history of thyroid cancer diagnosed any time—either before or after the diagnosis of breast cancer; **BC-1st**, breast cancer was diagnosed first, followed by diagnosis of thyroid cancer; **TC-1st**, thyroid cancer was diagnosed first, followed by diagnosis of breast cancer; **CI**, confident interval; **HR**, hazard ratio; **ER**, estrogen receptor; **PR**, progesterone receptor; **LN**, lymph node.

**Table G. Comparison of clinicopathological characteristics of breast cancer between patients also with a history of papillary thyroid cancer and patients with breast cancer only**

|  | **Comparison 1** | | | **Comparison 2** | | | **Comparison 3** | | |
| --- | --- | --- | --- | --- | --- | --- | --- | --- | --- |
|  | **BC/TC n/N(%)** | **BC-only n/N(%)** | **P value** | **BC-1st n/N(%)** | **BC-only n/N(%)** | **P value** | **TC-1st n/N(%)** | **BC-only n/N(%)** | **P value** |
| Number of cases | 2242 | 44,840 |  | 1063 | 24449 |  | 1179 | 31833 |  |
| Age at diagnosis(yrs)； Median (IQR) | 56 (48-66) | 56 (48-66) | 1.000 | 54 (46-62) | 54 (46-62) | 1.000 | 59 (40-69) | 59 (50-69) | 1.000 |
| Tumor Size(mm)；  Median (IQR) | 16 (10-25) | 17 (11-27) | <0.001 | 16 (10-25) | 18 (11-28) | 0.001 | 15 (10-25) | 17 (10-27) | <0.001 |
| Pathology Ductal | 1800/2242 (82.7) | 35890/44840 (83.3) | 0.798 | 873/1063 (85.0) | 19652/24449 (84.5) | 0.172 | 927/1150 (80.6) | 25292/31833 (81.9) | 0.359 |
| Lobular | 179/2242 (8.2) | 3367/44840 (7.8) | 0.429 | 73/1063 (7.1) | 1652/24449 (7.1) | 0.938 | 106/1150 (9.2) | 2607/31833 (8.4) | 0.233 |
| Mixed | 188/2242 (8.6) | 3532/44840 (8.2) | 0.406 | 75/1063 (7.3) | 1769/24449 (7.6) | 0.872 | 113/1150 (9.8) | 2784/31833 (9.0) | 0.223 |
| Inflammatory | 10/2242 (0.5) | 306/44840 (0.7) | 0.228 | 6/1063  (0.6) | 186/24449 (0.8) | 0.587 | 4/1150 (0.3) | 196/31833 (0.6) | 0.339 |
| ER/PR status ER-positive | 1371/1692 (81.0) | 26827/34367 (78.1) | 0.004 | 575/724 (79.4) | 12709/16837 (75.5) | 0.018 | 796/968 (82.2) | 21281/26654 (79.8) | 0.075 |
| R-positive | 1183/1671 (70.8) | 23184/34095 (70.0) | 0.018 | 498/712 (69.9) | 11034/16663 (66.2) | 0.043 | 685/959 (71.4) | 18345/26462 (69.3) | 0.176 |
| Both-positive | 1150/1671 (68.8) | 22465/34079 (65.9) | 0.016 | 480/712 (67.4) | 10589/16646 (63.6) | 0.043 | 670/959 (69.9) | 17870/26454 (67.6) | 0.140 |
| LN metastasis | 652/2001 (32.6) | 14442/40032 (36.1) | 0.002 | 301/905 (33.3) | 7973/20750 (38.4) | 0.002 | 351/1096 (32.0) | 10323/29743 (34.7) | 0.072 |
| Distant Metastasis | 68/2208 (3.1) | 6886/36997 (6.6) | <0.001 | 30/1044 (2.9) | 1679/23957 (7.0) | <0.001 | 38/1164 (3.3) | 2104/31364 (6.7) | <0.001 |
| AJCC Stage I+II | 1565/1824 (85.8) | 30111/36997 (81.4) | <0.001 | 667/783 (85.2) | 14807/18522 (79.9) | <0.001 | 898/1041 (86.3) | 23092/28099 (82.2) | <0.001 |
| III+IV | 259/1824 (14.2) | 6886/36997 (18.6) |  | 116/783 (14.8) | 3715/18522 (20.1) |  | 143/1041 (13.7) | 5007/28099 (17.8) |  |
| Radiation Therapy | 1076/2195 (49.0) | 22128/43676 (50.7) | 0.140 | 503/1046 (48.1) | 11419/23855 (47.9) | 0.914 | 573/1149 (49.9) | 16617/30932 (53.7) | 0.011 |
| BC-specific Mortality | 233/2242 (10.4) | 9368/44840 (20.9) | <0.001 | 96/1063 (9.0) | 6250/24449 (25.5) | <0.001 | 137/1179 (11.6) | 5233/31833 (16.4) | <0.001 |
| Follow Up Time；  Median ( (IQR) | 101  (44-188) | 79  (35-154) | <0.001 | 152  (79-235) | 101  (49-186) | <0.001 | 66  (28-134) | 63  (26-130) | 0.210 |
| With Insurance | 727/733  (99.2) | 14495/14679  (98.7) | 0.384 | 216/219  (98.6) | 4888/4971  (98.3) | 0.944 | 511/514  (99.4) | 13813/13990  (98.7) | 0.243 |
| Married | 1452/2154  (67.4) | 26912/43125  (62.4) | <0.001 | 705/1025  (68.8) | 15316/23617  (64.9) | 0.011 | 747/1129  (66.2) | 18389/30466  (60.4) | <0.001 |
| Unemployed * | 102/1343  (7.6) | 2091/26848  (7.8) | 0.837 | 37/526  (7.0) | 867/12088  (7.2) | 0.973 | 65/817  (8.0) | 1801/22051  (8.2) | 0.879 |
| Education (at lease bachelor degree * | 416/1343  (31.0) | 8426/26848  (31.4) | 0.776 | 159/526  (30.2) | 3674/12088  (30.4) | 0.974 | 258/817  (31.6) | 7024/22051  (31.9) | 0.899 |
| Median family income (US dollar; Median (IQR) | 65860  (51670-80800) | 65860  (52100-80620) | 0.922 | 60740  (46610-75740) | 60900  (48370-76350) | 0.518 | 66830  (53430-84180) | 66610  (53000-81810) | 0.493 |

**Notes**: Abbreviations: **BC**, breast cancer; **TC**, thyroid cancer; **BC-only**, patients only with breast cancer without a history of thyroid cancer; **BC/TC**, breast cancer patients also with a history of thyroid cancer diagnosed any time—either before or after the diagnosis of breast cancer; **BC-1st**, breast cancer was diagnosed first, followed by diagnosis of thyroid cancer; **TC-1st**, thyroid cancer was diagnosed first, followed by diagnosis of breast cancer; **IQR**, interquartile range; **ER**, estrogen receptor; **PR**, progesterone receptor; **LN**, lymph node. *The numbers of unemployed cases and cases with an education of least bachelor degree were obtained by calculation based on the unemployment rates and rates of education of at least bachelor degree and the corresponding total cases, respectively, in different counties

**Table H. Effects of a history of papillary thyroid cancer on breast cancer-specific mortality—deaths per 1000 person-years and hazard ratios**

|  |  | **BC-specific Mortality**  **n/N(%)** | **Deaths per 1000 Person-Years**  **(95% CI)** | **Unadjusted** | | | **Adjustment^a^** | | | **Adjustment^b^** | |
| --- | --- | --- | --- | --- | --- | --- | --- | --- | --- | --- | --- |
|  |  |  |  | **HR (95% CI)** | **P value** | **HR (95% CI)** | | **P value** | **HR (95% CI)** | | **P value** |
| Comparison 1 | BC-only | 9368/44840 (20.9) | 23.9(23.4-24.3) | 1.00 |  | 1.00 | |  | 1.00 | |  |
|  | BC/TC | 233/2242 (10.4) | 9.9 (8.7-11.2) | 0.42 (0.37-0.48) | <0.001 | 0.61 (0.50-0.70) | | <0.001 | 0.50 (0.39-0.66) | | <0.001 |
| Comparison 2 | BC-only | 6250/24449 (25.6) | 24.3 (23.7-25.0) | 1.00 |  | 1.00 | |  | 1.00 | |  |
|  | BC-1st | 96/1063 (9.0) | 6.5 (5.3-8.0) | 0.27 (0.22-0.34) | <0.001 | 0.41 (0.29-0.56) | | <0.001 | 0.25 (0.15-0.43) | | <0.001 |
| Comparison 3 | BC-only | 5233/31833 (16.4) | 22.6 (22.0-23.2) | 1.00 |  | 1.00 | |  | 1.00 | |  |
|  | TC-1st | 137/1179 (11.6) | 15.5 (13.0-18.4) | 0.68 (0.58-0.81) | <0.001 | 0.82 (0.64-1.05) | | 0.115 | 0.74 (0.55-1.01) | | 0.060 |

**Notes:** 1) Adjustment^a^ was made for race, tumor size, pathology type, ER/PR status, LN metastasis, radiation therapy, marital status and zip code. 2) Adjustment^b^ was made for race, tumor size, pathology type, ER/PR status, LN metastasis, radiation therapy, married status, employment status, education of at least bachelor degree, and median family income. 3) **BC**, breast cancer; **TC**, thyroid cancer; **BC-only**, patients only with breast cancer without a history of thyroid cancer; **BC/TC**, breast cancer patients also with a history of thyroid cancer diagnosed any time—either before or after the diagnosis of breast cancer; **BC-1st**, breast cancer was diagnosed first, followed by diagnosis of thyroid cancer; **TC-1st**, thyroid cancer was diagnosed first, followed by diagnosis of breast cancer; **CI**, confident interval; **HR**, hazard ratio; **ER**, estrogen receptor; **PR**, progesterone receptor; **LN**, lymph node.

**Table I. Differential protective effects of a history of papillary thyroid cancer on breast cancer-specific mortality in patients at age<50years and age≥50years at the diagnosis of breast cancer**

|  |  | **BC-specific Mortality**  **n/N(%)** | **Deaths per 1000 Person-Years**  **(95% CI）** | | **Unadjusted** | | **Adjustment^a^** | | **Adjustment^b^** | |
| --- | --- | --- | --- | --- | --- | --- | --- | --- | --- | --- |
|  |  |  |  |  | **HR (95% CI)** | **P Value** | **HR (95% CI)** | **P Value** | **HR (95% CI)** | **P Value** |
| Comparison 1 Age<50yrs | BC-only | 3492/13420 (26.0) | | 25.8 (25.0-26.7) | 1.00 |  | 1.00 |  | 1.00 |  |
|  | BC/TC | 69/671 (10.3) | | 7.5 (5.9-9.5) | 0.32 (0.25-0.41) | <0.001 | 0.37 (0.25-0.55) | <0.001 | 0.33 (0.20-0.56) | <0.001 |
| Comparison 1 Age>=50yrs | BC-only | 5876/31420 (18.7) | | 23.6 (23.0-24.3) | 1.00 |  | 1.00 |  | 1.00 |  |
|  | BC/TC | 164/1571 (10.4) | | 11.1 (9.6-13.0) | 0.49 (0.42-0.57) | <0.001 | 0.68 (0.54-0.86) | <0.001 | 0.54(0.40-0.74) | <0.001 |
| Comparison 2 Age<50yrs | BC-only | 2599/9016 (28.8) | | 26.8 (25.8-27.8) | 1.00 |  | 1.00 |  | 1.00 |  |
|  | BC-1st | 28/392 (7.1) | | 4.4 (3.0-6.4) | 0.19 (0.13-0.27) | <0.001 | 0.21 (0.11-0.39) | <0.001 | 0.12 (0.04-0.32) | <0.001 |
| Comparison 2 Age>=50yrs | BC-only | 3651/15433 (23.7) | | 24.7 (23.9-25.5) | 1.00 |  | 1.00 |  | 1.00 |  |
|  | BC-1st | 68/671 (10.1) | | 8.0 (6.3-10.2) | 0.34 (0.27-0.44) | <0.001 | 0.52 (0.35-0.75) | <0.001 | 0.31 (0.17-0.59) | <0.001 |
| Comparison 3 Age<50yrs | BC-only | 1537/7533 (20.4) | | 22.2 (21.1-23.3) | 1.00 |  | 1.00 |  | 1.00 |  |
|  | TC-1st | 41/279 (14.7) | | 14.6 (10.7-19.9) | 0.68 (0.50-0.93) | 0.015 | 0.83 (0.50-1.37) | 0.460 | 0.84 (0.46-1.54) | 0.581 |
| Comparison 3 Age>=50yrs | BC-only | 3696/24300 (15.2) | | 22.5 (21.8-23.3) | 1.00 |  | 1.00 |  | 1.00 |  |
|  | TC-1st | 96/900 (10.7) | | 15.5 (12.6-18.9) | 0.68 (0.55-0.84) | <0.001 | 0.81 (0.61-1.08) | 0.147 | 0.68 (0.48-0.98) | 0.036 |

**Notes:** 1) Adjustment^a^ was made for race, tumor size, pathology type, ER/PR status, LN metastasis, radiation therapy, marital status and zip code. 2) Adjustment^b^ was made for race, tumor size, pathology type, ER/PR status, LN metastasis, radiation therapy, married status, employment status, education of at least bachelor degree and median family income. 3) Abbreviations: **BC**, breast cancer; **TC**, thyroid cancer; **BC-only**, patients only with breast cancer without a history of thyroid cancer; **BC/TC**, breast cancer patients also with a history of thyroid cancer diagnosed any time—either before or after the diagnosis of breast cancer; **BC-1st**, breast cancer was diagnosed first, followed by diagnosis of thyroid cancer; **TC-1st**, thyroid cancer was diagnosed first, followed by diagnosis of breast cancer; **CI**, confident interval; **HR**, hazard ratio; **ER**, estrogen receptor; **PR**, progesterone receptor; **LN**, lymph node.

**Table J. Comparison of ER/PR expression status in breast cancer between patients also with a history of thyroid cancer and patients only with breast cancer** **at age <50 years or age** **≥50 years**

|  | **Age** | **ER-PR status** | **BC/TC n/N(%)** | **BC-only n/N(%)** | **P** |
| --- | --- | --- | --- | --- | --- |
| Case Selection 1 | <50 | ER positive | 384/498 (77.1) | 7350/10234 (71.8) | 0.012 |
|  |  | PR positive | 353/491 (71.9) | 6695/10150 (66.0) | 0.008 |
|  | ≥50 | ER positive | 1167/1403 (83.2) | 22785/28267 (80.6) | 0.019 |
|  |  | PR positive | 981/1386 (70.8) | 19246/28044 (68.6) | 0.098 |
| Case Selection 2 | <50 | ER positive | 384/498 (77.1) | 7356/10215 (72.0) | 0.015 |
|  |  | PR positive | 353/491 (71.9) | 6791/10133 (67.0) | 0.028 |
|  | ≥50 | ER positive | 1167/1403 (83.2) | 22753/28333 (80.3) | 0.009 |
|  |  | PR positive | 981/1386 (70.8) | 19217/28079 (68.4) | 0.072 |
| Case Selection 3 | <50 | ER positive | 384/498 (77.1) | 7314/10229 (71.5) | 0.008 |
|  |  | PR positive | 353/491 (71.9) | 6723/10142 (66.3) | 0.012 |
|  | ≥50 | ER positive | 1167/1403 (83.2) | 22827/28365 (80.5) | 0.014 |
|  |  | PR positive | 981/1386 (70.8) | 19322/28134 (68.7) | 0.106 |
| White Patients | <50 | ER positive | 277/353 (78.5) | 5294/7287 (72.6) | 0.019 |
|  |  | PR positive | 250/345 (72.5) | 4913/7211 (68.1) | 0.103 |
|  | ≥50 | ER positive | 928/1096 (84.7) | 18003/22039 (81.7) | 0.014 |
|  |  | PR positive | 785/1081 (72.6) | 15275/21853 (69.9) | 0.061 |
| PTC | <50 | ER positive | 356/465 (76.6) | 6737/9444 (71.3) | 0.017 |
|  |  | PR positive | 326/457 (71.3) | 6154/9353 (65.8) | 0.017 |
|  | ≥50 | ER positive | 1015/1227 (82.7) | 20090/24923 (80.6) | 0.073 |
|  |  | PR positive | 857/1214 (70.6) | 17030/24742 (68.8) | 0.206 |

**Abbreviations: BC**, breast cancer; **TC**, thyroid cancer; **BC/TC**, breast cancer patients also with a history of thyroid cancer diagnosed any time—either before or after the diagnosis of breast cancer; **BC-only**, patients only with breast cancer without a history of thyroid cancer; **PTC**, papillary thyroid cancer.
